# P2′ Site Scanning of Snake Kunitz-Type Toxin Analogue BF9-N17H with Unique P1 Site Histidine Highlights Novel XIa Inhibitory Peptides Without Plasmin Inhibitory Activity

**DOI:** 10.3390/toxins18100449

**Published:** 2026-10-15

**Authors:** Luyao Wang, Li Ding, Tianxiang Qin, Xinyu Liu, Chenglan Lou, Hongyi Luo, Fang Sun, Chenhu Qin, Xiangdong Ye, Xudong Luo, Zongyun Chen

**Affiliations:** 1 Hubei Key Laboratory of Embryonic Stem Cell Research and Hubei Key Laboratory of Wudang Local Chinese Medicine, Department of Biochemistry and Molecular Biology, Research of Institute of Biomedicine, College of Basic Medicine, Hubei University of Medicine, Shiyan 442000, China; 2 Department of Clinical Laboratory, Dongfeng Hospital, Hubei University of Medicine, Shiyan 442008, China

**Keywords:** XIa inhibitor, Kunitz-type peptide, snake toxin peptide, BF9-N17H, plasmin inhibitor

## Abstract

Kunitz-type peptides from venomous and blood-sucking animals represent an important natural molecular resource for anticoagulant development towards XIa, a new target for antithrombotic drug discovery. However, the plasmin-inhibitory activity of these Kunitz-type XIa inhibitory anticoagulants limits their application. Here, using the snake Kunitz-type toxin analogue BF9-N17H, an XIa inhibitor with a unique P1-site histidine, as a template, P2′ scanning was performed to enhance its selectivity towards XIa. Ten BF9-N17H analogues were designed using different amino acid classification replacement at the P2′ site, including non-polar amino acids A and P, acidic amino acids D and E, polar amino acids S, N, and Y, and basic amino acids K, R, and H. Coagulation factor inhibitory experiments showed that three BF9-N17H analogues, BF9N17HP2, BF9N17HK8 and BF9N17HR9, lost their XIa inhibitory activity, but seven other analogues, BF9N17HA1, BF9N17HD2, BF9N17HS4, BF9N17HN5, BF9N17HY6, BF9N17HE7 and BF9N17HH10, effectively maintained their inhibitory activity towards XIa, with IC_50_ values from 61.19 ± 7.04 nM to 4.54 ± 3.65 nM. One analogue, BF9N17HD2, inhibited XIa with an IC_50_ of 6.19 ± 1.38 nM and had a selectivity index of 142.79 towards plasmin. It only had weak inhibitory activity toward plasmin, with an IC_50_ of 883.88 ± 148.35 nM. Excitingly, five analogues, BF9N17HS4, BF9N17HN5, BF9N17HY6, BF9N17HE7 and BF9N17HH10, almost lost their plasmin-inhibitory activity completely, indicating their high selectivity towards XIa. To the best of our knowledge, this is the first report of Kunitz-type XIa inhibitors without plasmin-inhibitory activity, and it highlights that the Kunitz-type peptide scaffold might be used as a potential candidate for the development of potent and selective XIa-inhibitory antithrombotic agents.

## 1. Introduction

Coagulation factor XIa is a new drug target that belongs to the classical serine–protease family for new-generation anticoagulant drugs, which mainly plays a key role in the contact activation stage and might have low bleeding risk and favourable antithrombotic activity [1,2,3,4,5]. With the successful discovery of the specific anticoagulants targeting Xa and IIa, the new generation-specific anticoagulants targeting XIa are thought to have broad prospects and huge clinical value [3]. During the Xa- and IIa-targeting anticoagulant development, bioactive peptides and their analogues from venomous animals and blood-sucking animals provide valuable leads for clinical drug discovery, such as tick-derived Kunitz-type peptide TAP and its analogues and leech-derived hirudin and its analogues [6,7,8,9,10,11]. So, it is very important to identify diverse natural peptides and peptide analogues with potent and selective XIa inhibitory activity for the further lead discovery of clinical anticoagulant drugs.

The Kunitz-type domain constitutes a natural peptide scaffold characterized by a well-defined, stable fold stabilized by three conserved disulfide bonds. Widely present across diverse animal taxa, it serves as a highly valuable molecular template to research and design XIa inhibitors [12,13]. Several potent Kunitz-type XIa inhibitors have been identified, such as the snake *Bungarus fasciatus*-derived BF9 and Fasxiator [14,15,16], the human protease nexin 2-derived PN2KPI [17,18,19], the Leech *Whitmania pigra*-derived WPK5 [20], the snake *Deinagkistrodon acutus*-derived DAKS1 [21], the tick *Ixodes Ricinus* Ir-CPI [22], and the vampire bat *Desmodus rotundus*-derived Desmolaris [13]. In addition to this, several artificially engineered Kunitz-type peptides with enhanced XIa inhibitory selectivity have been generated, such as Fasxiator_N17R,L19E_, DX-88mut, PN2KPI-M17D and WPK5-mut [14,20,23,24]. However, sequence analyses indicated that all these Kunitz-type XIa inhibitors with high activity and enhanced selectivity had a common P1 site R, except two weak anticoagulants and XIa-inhibitory snake venom Kunitz-type peptides BF9 and Fasxiator with a P1 site N. To date, no studies have described selectivity-focused optimization of Kunitz-type XIa inhibitors possessing a histidine at the P1 position [25,26].

In our previous work, we generated a panel of BF9 analogues (snake Kunitz-type toxin) via P1-site scanning mutagenesis using residue-class-based substitution and found that the replacement of basic amino acids K, R and H at the P1 site apparently enhanced their XIa inhibitory activity [14,15]. Among them, BF9-N17H is the first XIa inhibitor with a unique P1 site histidine [14]. In this work, we further performed residue-class-directed substitutions at the P2′ site using BF9-N17H as the parental template. This set of experiments aimed to assess how the P2′ residue modulates the inhibitory potency and target selectivity of Kunitz-scaffold peptides toward factor XIa, a promising target for next-generation antithrombotic therapeutics.

## 2. Results

### 2.1. Molecular Design and Functional Expression of Ten BF9-N17H Analogues with the P2′-Site Substitution

**Figure 1:**
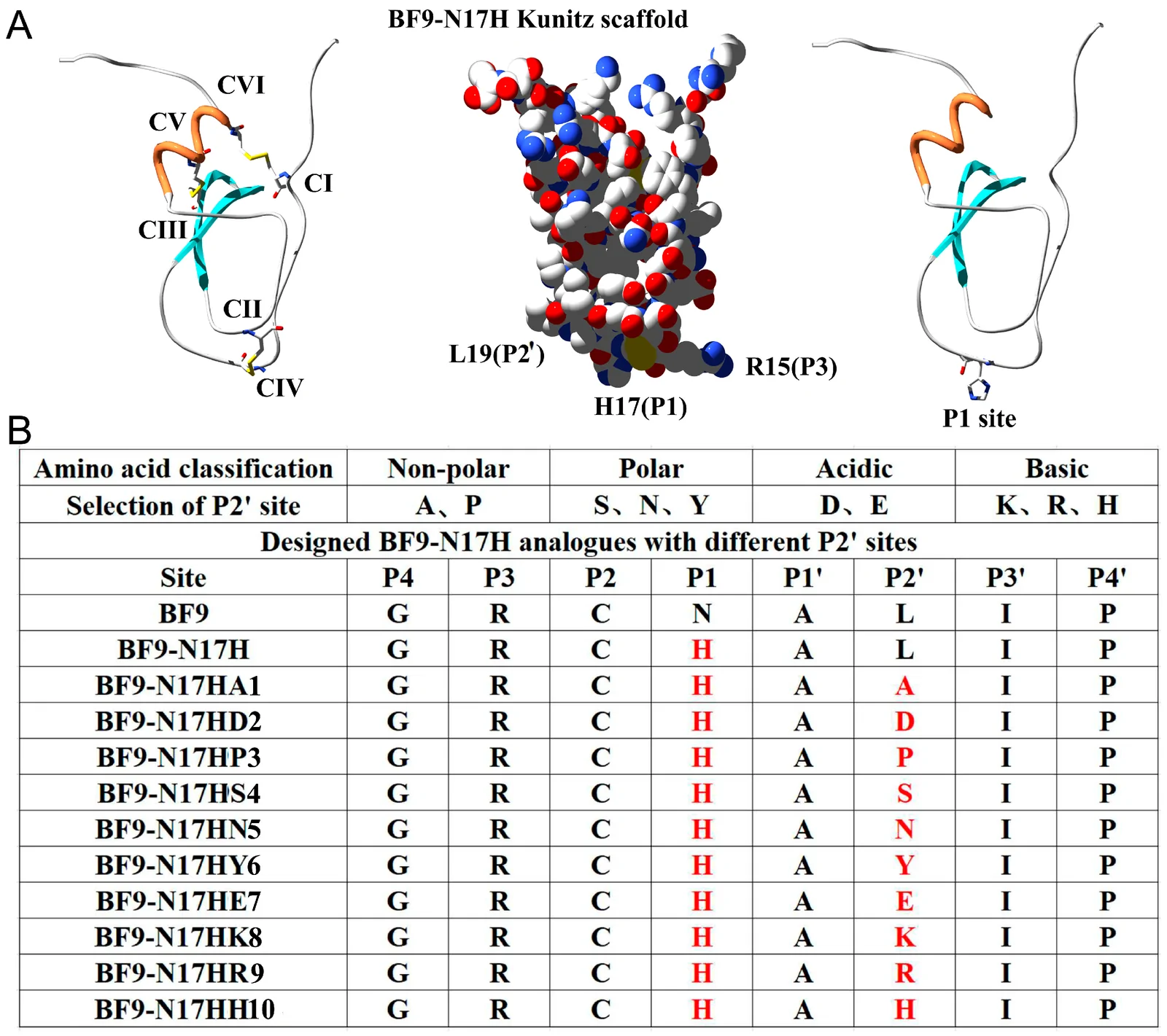
P2′ site amino acid substitution strategy based on the Kunitz-scaffold and canonical amino acid classification. (**A**) Snake toxin analogue BF9-N17H was selected as a template. (**B**) Key sequence comparison of ten BF9-N17H analogues with different kinds of amino acid replacements in the P2′ site.

BF9 is a Kunitz-type peptide from the venom of the snake *Bungarus fasciatus*, and it adopts a compact pear-shaped conformation stabilized by three conserved disulfide bonds [16]. So, snake venom-derived peptide BF9 and its analogues are very suitable for peptide engineering and represent excellent non-antibody scaffolds [14,27,28,29,30]. In our previous work, we generated a panel of BF9 variants by changing the P1 site and discovered the first XIa inhibitor BF9-N17H with the P1 site histidine. However, the plasmin inhibitory activity of BF9-N17H limits its utility as a selective anticoagulant lead compound [14]. In order to further enhance the selectivity of BF9-N17H towards XIa, ten new BF9-N17H analogues were designed by using the P2′ site replacement strategy based on amino acid classification, including polar amino acids S, N, and Y, non-polar amino acids A and P, basic amino acids K, R, and H, and acidic amino acids D and E (Figure 1). These designed peptides, BF9N17HA1, BF9N17HP2, BF9N17HD3, BF9N17HS4, BF9N17HN5, BF9N17HY6, BF9N17HE7, BF9N17HK8, BF9N17HR9 and BF9N17HH10, were expressed and purified following protocols previously established [14,15,16]. All these designed peptides were also formed into inclusion bodies in *Escherichia coli* and were refolded in the GSH-GSSG system that we have used in the expression system of the template peptide BF9-N17H. The folded peptides were purified by HPLC and collected manually (Figure S1). Peptides were finally purified by RP-HPLC on a C18 column (i.d. 10 mm, length 250 mm, particle size 5 μm, Elite-HPLC) using a linear gradient from 5% to 95% acetonitrile with 0.1% TFA in 45 min or 60 min with a constant flow rate of 5 mL/min. Peaks were monitored at 230 nm. All the recombinant peptides were stored at −20 °C after quantitative identification with SDS-PAGE (Figure S2).

In order to confirm the functional expression of ten BF9-N17H analogues, we first profiled their inhibitory activity for three serine proteases from the digestive system, trypsin, chymotrypsin and elastase. It was found that ten designed peptides exhibited inhibition profiles comparable to the template Kunitz-type peptide BF9-N17H, suggesting that they were expressed functionally (Figures S3–S5). All ten engineered peptides showed no inhibitory activity towards elastase and had weak trypsin inhibitory activity (Figures S3 and S4). For chymotrypsin, the inhibitory activities of ten designed peptides were different. Seven analogues, BF9-N17HA1, BF9-N17HS4, BF9-N17HN5, BF9-N17HY6, BF9-N17HK8, BF9-N17HR9 and BF9-N17HH10, showed apparent inhibitory activity towards chymotrypsin, one analogue, BF9N17HE7, showed weak chymotrypsin inhibitory activity, and two analogues, BF9N17HD3 and BF9N17HP2, showed no activity towards chymotrypsin. The inhibitory profiles towards three digestive serine proteases indicated their functional expressions of ten designed peptides and could be used for further coagulation factor inhibitory scanning.

### 2.2. Discovery of One XIa Inhibitor with Weak Plasmin Inhibitory Activity and Five XIa Inhibitors Without Plasmin Inhibitory Activity

**Figure 2:**
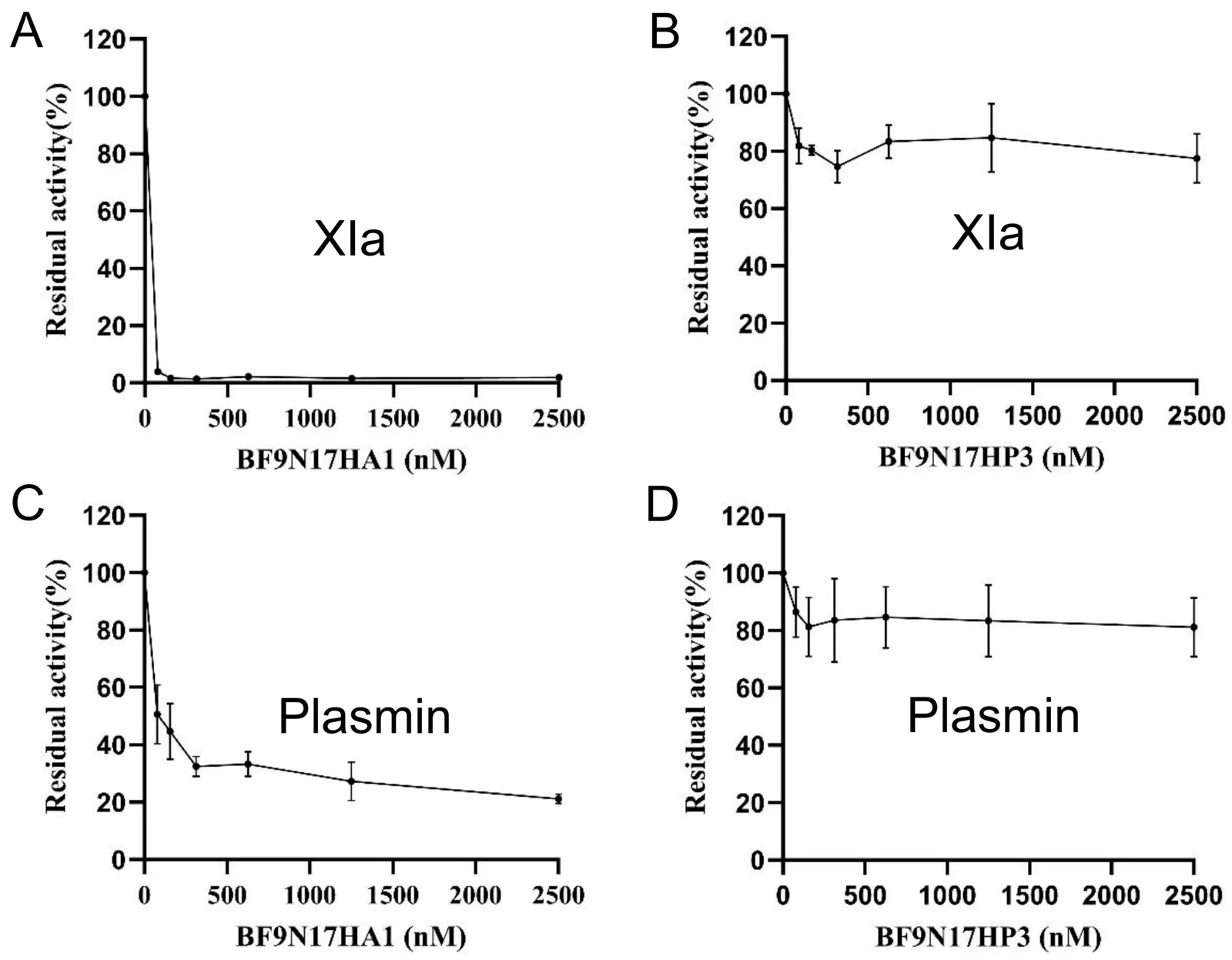
XIa and plasmin inhibitory activities of BF9-N17H P2′ site scanning peptides with non-polar amino acids A and P. (**A**) Inhibitory activity of BF9N17HA1 towards XIa; (**B**) inhibitory activity of BF9N17HP3 towards XIa; (**C**) plasmin inhibitory activity of BF9N17HA1; (**D**) plasmin inhibitory activity of BF9N17HP3.

**Figure 3:**
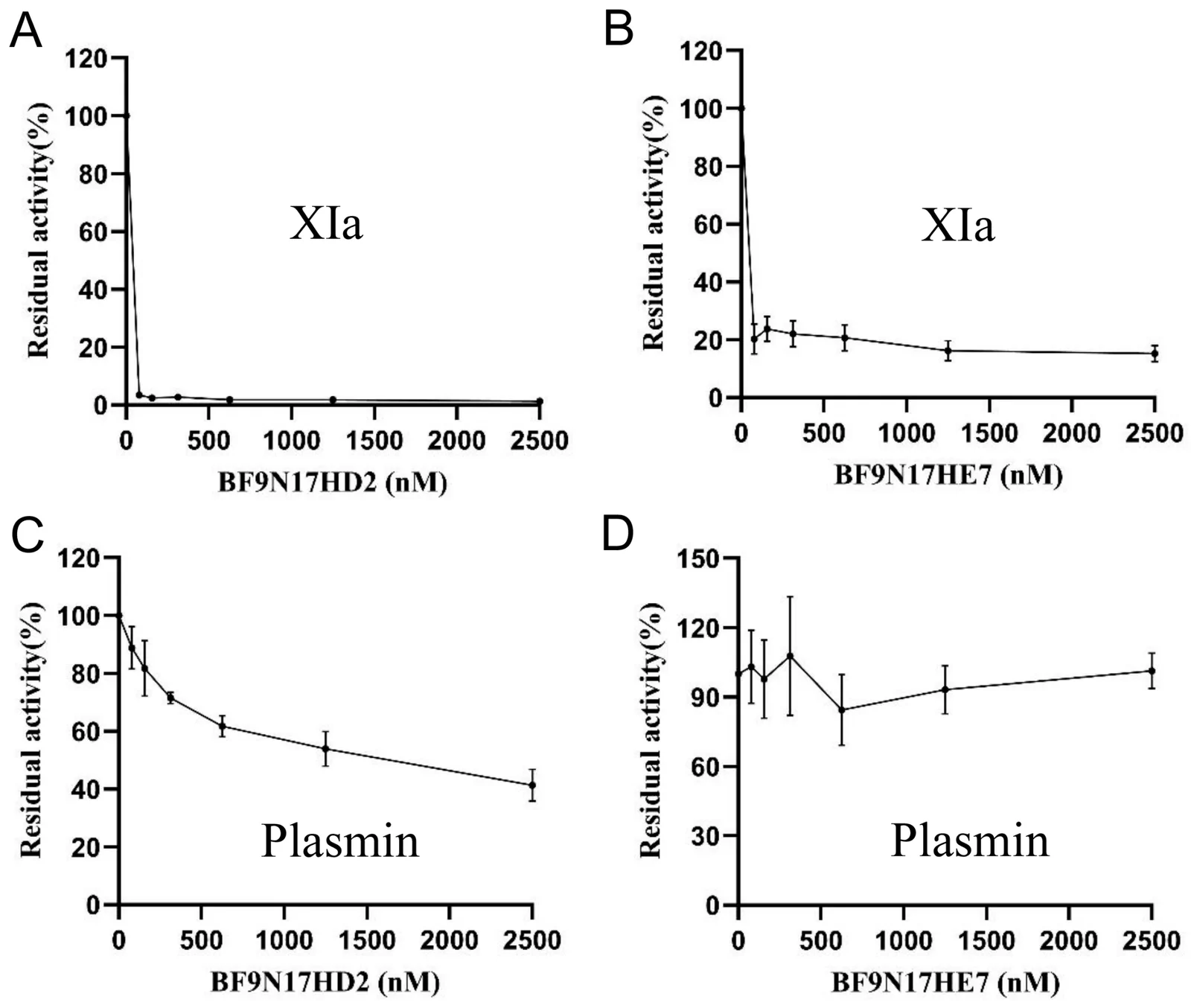
XIa and plasmin inhibitory activities of BF9-N17H P2′ site scanning peptides with acidic amino acids D and E. (**A**) The inhibitory activity of BF9N17HD2 for XIa; (**B**) the inhibitory activity of BF9N17HE7 for XIa; (**C**) plasmin inhibitory activity of BF9N17HD2; (**D**) plasmin inhibitory activity of BF9N17HE7.

**Figure 4:**
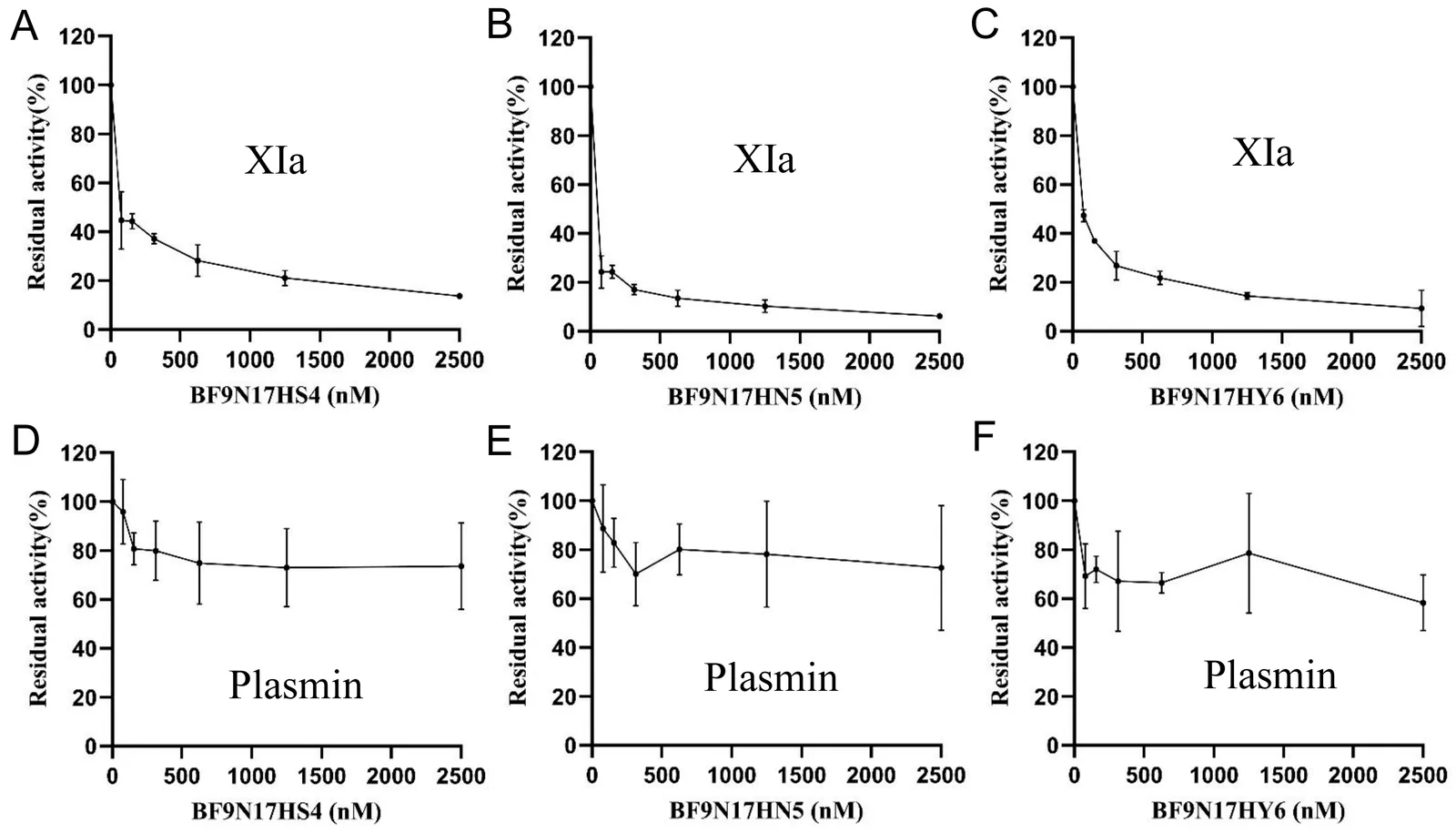
XIa and plasmin inhibitory activities of BF9-N17H P2′ site scanning peptides with polar amino acids S, N and Y. (**A**) XIa inhibitory effect of BF9N17HS4; (**B**) XIa inhibitory effect of BF9N17HN5; (**C**) XIa inhibitory effect of BF9N17HY6; (**D**) plasmin inhibitory activity of BF9N17HS4; (**E**) plasmin inhibitory activity of BF9N17HN5; (**F**) plasmin inhibitory activity of BF9N17HY6.

**Figure 5:**
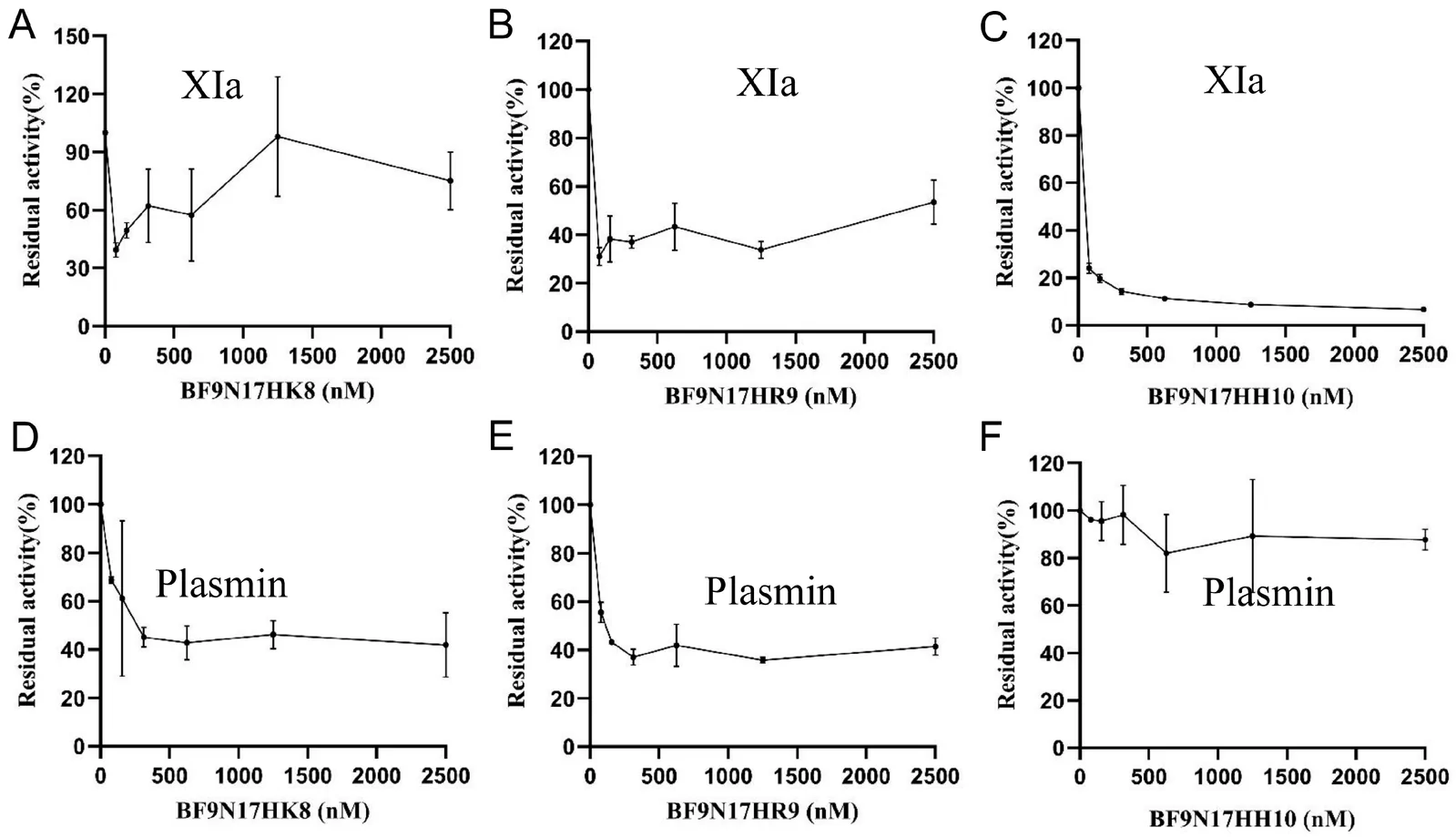
XIa and plasmin inhibitory activities of BF9-N17H P2′ site scanning peptides with basic amino acids K, R and H. (**A**) XIa inhibitory function of BF9N17HK8; (**B**) XIa inhibitory function of BF9N17HR9; (**C**) XIa inhibitory function of BF9N17HH10; (**D**) plasmin inhibitory activity of BF9N17HK8; (**E**) plasmin inhibitory activity of BF9N17HR9; (**F**) plasmin inhibitory activity of BF9N17HH10.

For the functional evaluation of these P2′ site mutant peptides BF9N17HA1, BF9N17HP2, BF9N17HD3, BF9N17HS4, BF9N17HN5, BF9N17HY6, BF9N17HE7, BF9N17HK8, BF9N17HR9 and BF9N17HH10, the inhibitory activities towards XIa and plasmin were tested first. For the replacement of two non-polar amino acids, A and P (Figure 2), it was found that one analogue, BF9N17HA1, had apparent XIa and plasmin inhibitory activities with IC_50_ values of 6.67 ± 2.19 nM and 48.72 ± 8.67 nM, respectively, and another analogue, BF9N17HP2, showed no apparent XIa or plasmin inhibitory activities. For the replacement of two acidic amino acids, D and E (Figure 3), both analogues BF9N17HD2 and BF9N17HE7 showed apparent inhibitory activities towards Xia, with IC_50_ values of 6.19 ± 1.34 nM and 4.54 ± 3.65 nM, respectively. In addition to this, the maximal inhibition of BF9N17HD2 was better than BF9N17HE7. BF9N17HE7 had lost the inhibition effect towards plasmin, but BF9N17HD2 still had plasmin inhibitory activity with an IC_50_ value of 883.87 ± 148.35 nM. For replacement of the three polar amino acids S, N and Y (Figure 4), their inhibitory profiles towards both XIa and plasmin were similar. BF9N17HS4, BF9N17HN5 and BF9N17HY6 had apparent inhibitory activities towards Xia, with IC_50_ values of 54.04 ± 16.40 nM, 19.96 ± 5.15 nM and 61.19 ± 7.04 nM, respectively. All three analogues with polar amino acid replacement at the P2′ site had lost their plasmin inhibitory activities, which suggested that polar amino acid replacement might be a useful choice to enhance Kunitz-type peptide selectivity towards XIa when its P1 site is histidine. For the replacement of three basic amino acids K, R and H (Figure 5), the inhibitory profiles were complex and different. BF9N17HK8 and BF9N17HR9 lost their XIa inhibitory activities, but BF9N17HH10 still had apparent XIa inhibitory activity, with an IC_50_ value of 18.75 ± 2.34 nM. Conversely, BF9N17HH10 lost its plasmin inhibitory activity, but BF9N17HK8 and BF9N17HR9 still had some plasmin inhibitory activities, with IC_50_ values of 69.58 ± 15.44 nM and 26.09 ± 8.74 nM, respectively.

**Figure 6:**
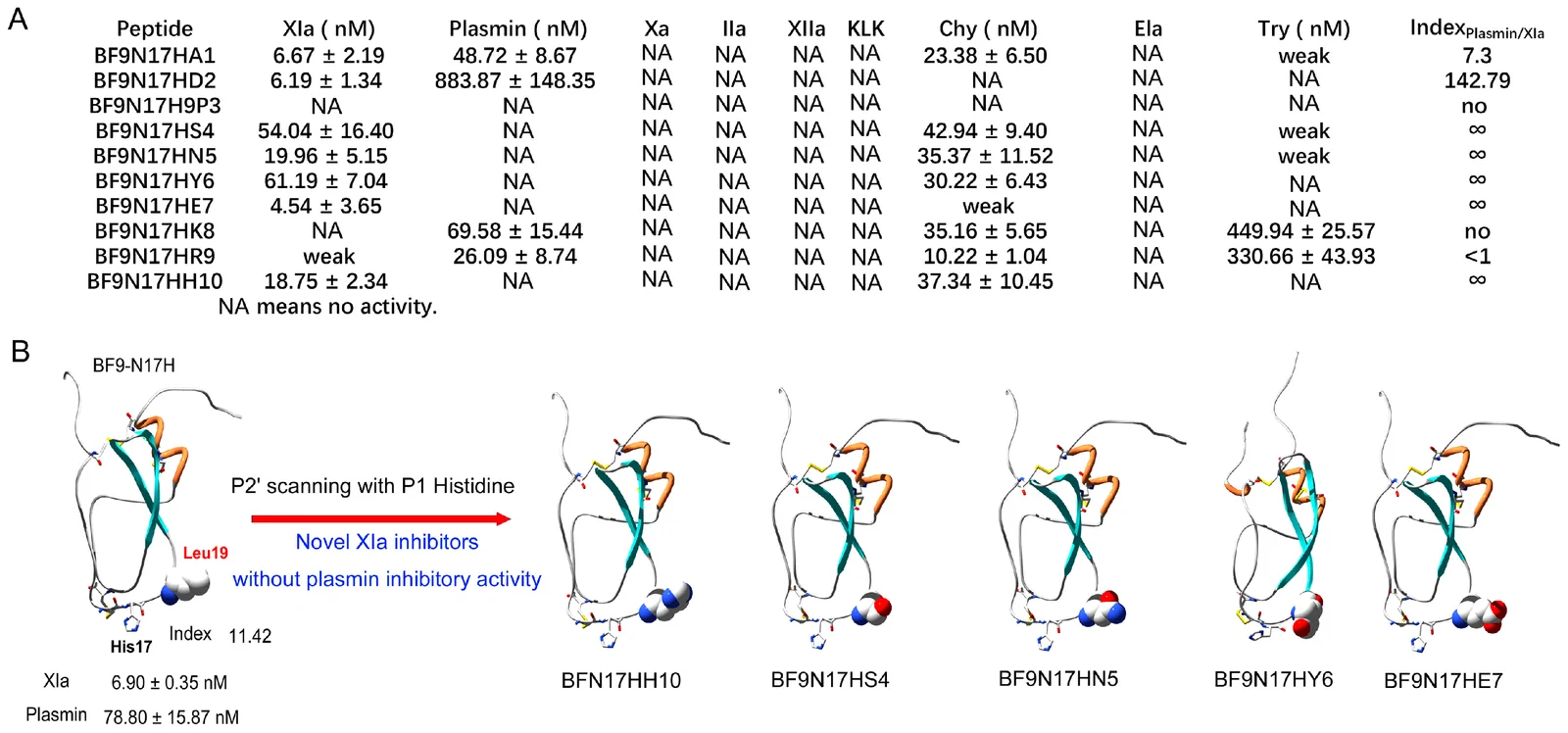
Serine protease inhibitory activity comparison highlights five novel XIa inhibitors without plasmin inhibitory activity. (**A**) Inhibitory effect comparison of ten BF9-N17H analogues with their IC_50_ values towards different enzymes. XIa, Xa, IIa and XIIa are different coagulation factors, KLK is plasma kallikrein, Chy is chymotrypsin, Ela is elastase, and Try is trypsin. (**B**) Five XIa inhibitors without plasmin inhibitory activity.

These results toward XIa and plasmin showed that the seven analogues BF9N17HA1, BF9N17HD3, BF9N17HS4, BF9N17HN5, BF9N17HY6, BF9N17HE7 and BF9N17HH10 still had apparent inhibitory activities towards XIa. Three analogues, BF9N17HP2, BF9N17HK8 and BF9N17HR9, lost their XIa inhibitory activities completely. Among them, BF9N17HA1 and BF9N17HD3 had similar inhibitory activity towards Xia, with IC_50_ values of 6.67 ± 2.19 nM and 6.19 ± 1.38 nM, respectively. However, BF9N17HA1 exerted the strongest plasmin inhibition among all ten variants. Although BF9N17HD3 still had weak plasmin inhibitory activity with an IC_50_ of 883.88 ± 148.35 nM, it is weaker than BF9N17HA1, suggesting appreciable selectivity for XIa. These results indicated that by the P2′ site replacement strategy, we discovered a potent and selective XIa inhibitor, BF9N17HD3, with a unique P1 site histidine and a good selection index of 142.79 (Figure 6). Excitingly, the five XIa inhibitory analogues BF9N17HS4, BF9N17HSN5, BF9N17HY6, BF9N17HE7 and BF9N17HH10 showed no plasmin inhibitory activities. This finding establishes a new design route toward plasmin-sparing XIa-targeted inhibitors.

Xa and IIa are two classical anticoagulant targets for some clinically approved anticoagulants including rivaroxaban, apixaban, dabigatran, and bivalirudin. So, we further tested the inhibitory activities of ten engineered peptides towards Xa and IIa. The results showed that all ten engineered peptides showed no inhibitory activity towards Xa and IIa, which was consistent with the parental BF9-N17H scaffold (Figures S6 and S7). This observation confirms that snake venom Kunitz-type peptide BF9 and its analogues are a good molecular scaffold to design selective XIa inhibitors with no Xa or IIa inhibitory activities. By contrast, all ten engineered peptides had no inhibitory activity towards the coagulation factors XIIa and KLK (plasma kallikrein) in the intrinsic coagulation pathway (Figures S8 and S9), which was also consistent with the template peptide serine protease activity profiles. In summary, by the P2′ site scanning strategy and serine protease inhibitory activity assay, we discovered one XIa inhibitor with weak plasmin inhibitory activity and five XIa inhibitors without plasmin inhibitory activity, which showed a good selection index towards haemostatic target plasmin and also had no inhibitory activity towards the other coagulation factors Xa, IIa, XIIa and KLK.

### 2.3. Structure and Functional Relationship Analyses of BF9-N17H Analogues That Selectively Inhibited XIa

Through molecular design of the P2′ site replacement and functional scanning, we identified one XIa inhibitor with weak plasmin inhibitory activity, BF9N17HD2, and five XIa inhibitors, BF9N17HS4, BF9N17HSN5, BF9N17HY6, BF9N17HE7 and BF9N17HH10, without plasmin inhibitory activity. Considering that there is only one amino acid difference between BF9-N17H and its analogues at the P2′ site, we performed a structure–activity relationship (SAR) analysis of Kunitz-type peptide BF9-N17H analogues that inhibited XIa and plasmin by comparing their inhibitory activities with their primary structures (Figure 6).

For XIa, it was found that the P2′ site P, K or R of the peptide almost abolished its XIa inhibitory activity, and the P2′ site A, D, S, N, H, Y or E of the peptide still retained different degrees of inhibitory activity towards XIa. These results suggested that strongly basic amino acids K and R in the P2′ site might not be suitable for XIa-inhibitory Kunitz-type peptides, but weakly basic amino acid H is different from K and R because BF9N17HH10 still had apparent XIa inhibitory activity. In addition to this, acidic amino acids, polar amino acids and non-polar acids are also suitable for XIa-inhibitory Kunitz-type peptides in our system (Figure 6A). In summary, substitution of the P2′ position with non-polar amino acids, polar acids, acidic acids and weakly basic residue H represents a viable strategy to retain or improve the XIa inhibitory activity of Kunitz-type peptides with the P1 site histidine.

Plasmin also is a proteolytic enzyme in blood plasma that dissolves the fibrin in blood clots. For specific anticoagulant discovery, plasmin is a haemostatic side effect target. For plasmin, the P2′ site amino acids P, S, N, H, Y or E of the peptide almost abolished the peptide inhibitory activity towards plasmin, but the P2′ site amino acids A, D, K and R still had different degrees of inhibitory activity towards plasmin. These analyses suggested that the P1 site histidine and the P2′ site S, N, H, Y or E might be potential choices for selective Kunitz-type XIa inhibitors without plasmin inhibitory activity (Figure 6B).

## 3. Discussion

It is widely accepted that sufficient target selectivity is a prerequisite for therapeutic efficacy and minimizing off-target adverse effects of any drug-candidate molecule. Favourable pharmacological performance serves as an essential prerequisite and driving force for pharmacokinetic optimization. Hence, improving the selectivity of Kunitz-type XIa peptide inhibitors carries great significance and represents a core bottleneck determining whether the Kunitz scaffold can be engineered into therapeutic XIa-targeting anticoagulant peptides. In our present work, five Kunitz-type peptide analogues carrying a P1-site histidine, BF9N17HS4, BF9N17HN5, BF9N17HY6, BF9N17HE7 and BF9N17HH10, displayed a near-complete loss of plasmin-inhibitory activity but retained their XIa inhibitory activities, indicating their high selectivity towards XIa. To the best of our knowledge, this constitutes the first report of Kunitz-type XIa inhibitors without plasmin inhibitory activity and highlights that the Kunitz-type peptide scaffold holds promise as a template for the discovery of specific XIa inhibitors. These results also indicated the unique pharmacological effects of Kunitz-type peptides with the P1 site histidine and underscore the combinatorial mutational effect conferred by P1-site histidine (instead of lysine or arginine) and the P2′ site scanning for the discovery of XIa inhibitors without plasmin inhibitory activity.

**Figure 7:**
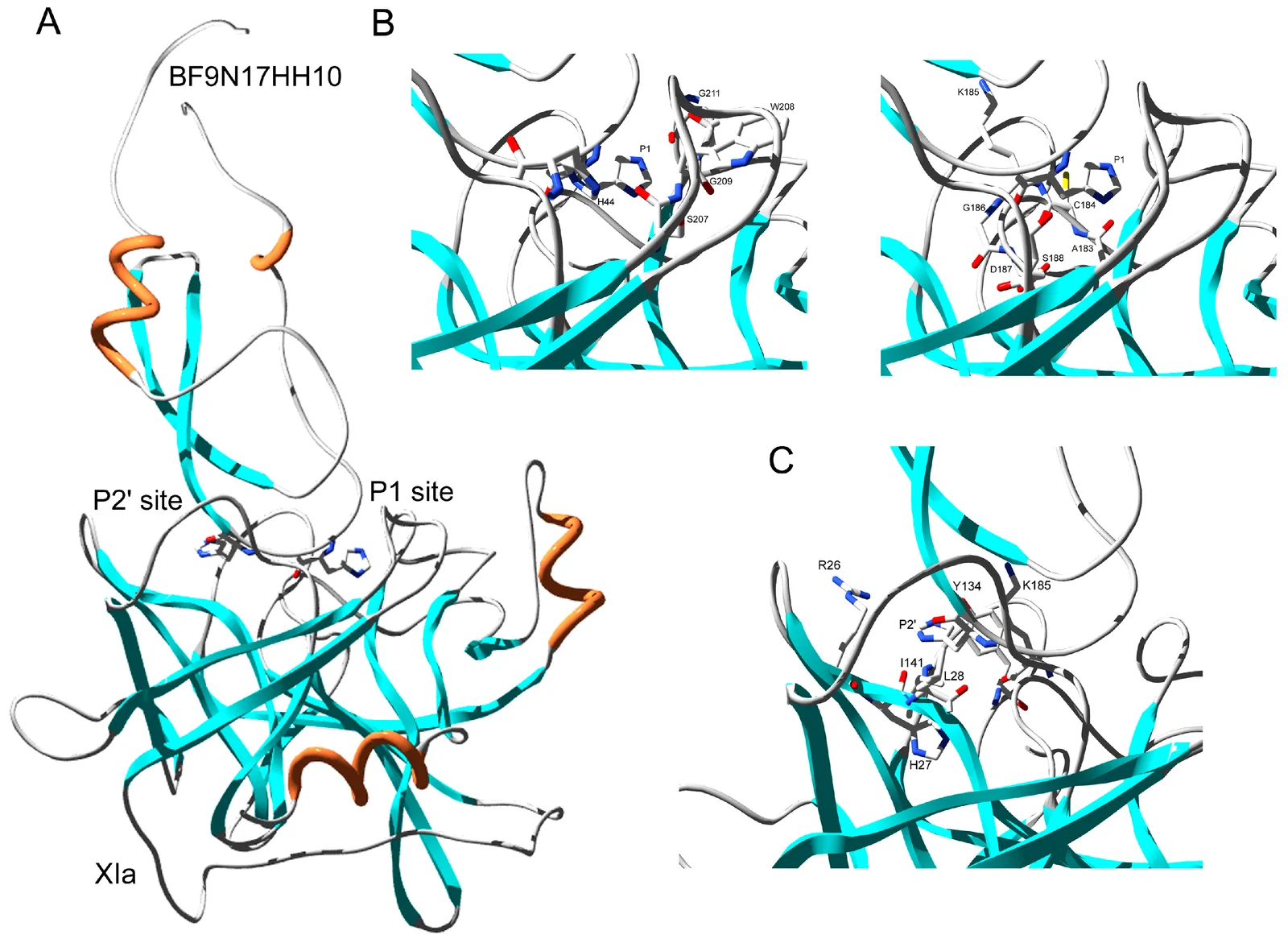
Interaction mechanisms of one analogue, BF9N17HH10, with coagulation factor XIa. (**A**) BF9N17HH10 and XIa complex structure was explored by α-fold3; (**B**) key residues of XIa interacting with P1 site histidine of BF9N17HH10; (**C**) key residues of XIa interacting with P2′ site histidine of BF9N17HH10.

The role of the three amino acids K, R, and H at the P1 site and the P2′ site of Kunitz-type peptides might be completely different, and combinatorial mutation effects between two positions might also be different. BF9N17HH10 had apparent inhibitory activity towards XIa, but BF9N17HK8 and BF9N17HR9 only showed weak inhibitory activity towards XIa. BF9N17HK8 and BF9N17HR9 with the lysine and arginine replacements at the P2′ site are two inhibitors for haemostatic target plasmin, but BF9N17HH10 with double histidine at the P1 site and the P2′ site is a selective inhibitor for anticoagulation target XIa without plasmin inhibitory activity (Figure 6). We built a structural model of the BF9N17HH10-XIa complex using AlphaFold3 (Figure 7A). For the P1 site histidine of BF9N17HH10, interacting residues on XIa were mapped to three surface regions, H44, A183, C184, R185, G186, D187, S188, S207, W208, G209 and G211 (Figure 7B). For the P2′ site histidine of BF9N17HH10, the interacted residues of XIa are dispersed, which are R26, H27, L28, Y134, I141 and K185 (Figure 7C). These results showed that the functional role of three basic residues in the peptides might be very complex, and they might have similar or opposite biological function in different situations [31,32]. Although lysine, arginine, and histidine are all basic residues positively charged under physiological pH, they possess chemically distinct side-chains: an amino group (Lys), a guanidino group (Arg), and an imidazole group (His). So, it is very important to scan the three basic amino acids completely for the protein and peptide engineering in the future [33,34,35].

**Figure 8:**
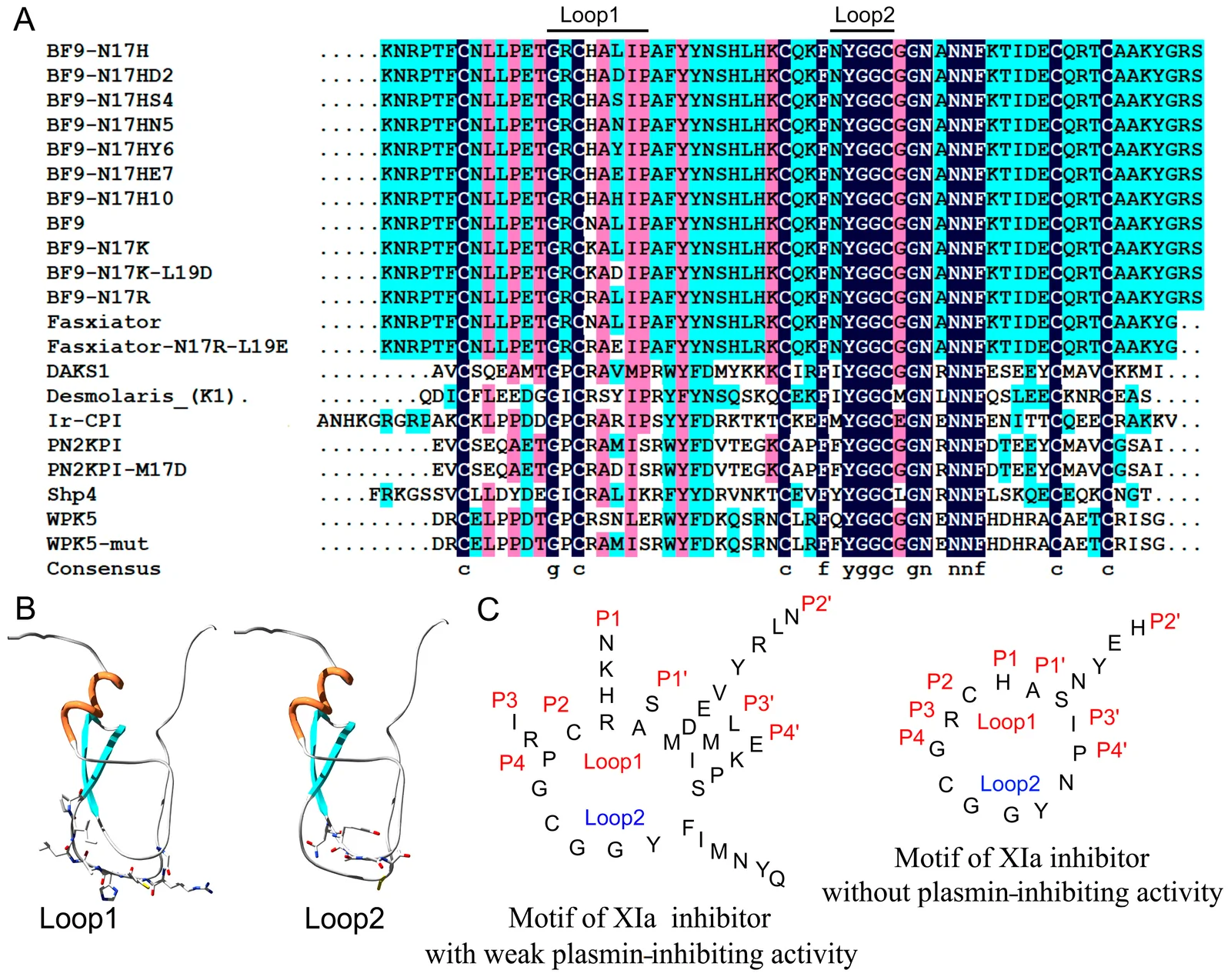
Functional motif comparison of XIa Kunitz-type inhibitors. (**A**) Sequence alignments of all the known Kunitz-type XIa inhibitors; (**B**) potential functional motifs of XIa inhibitors with Kunitz-type structural fold; (**C**) motif visualization of Kunitz-type XIa inhibitors with weak plasmin inhibitory activity and Kunitz-type XIa inhibitors without plasmin inhibitory activity.

As we know, Kunitz-type XIa inhibitors have been reported by multiple research groups and from different animals (Figure 8), such as BF9 and Fasxiator [15,16,27], PN2KPI [19,36], WPK5 [20], DAKS1 [21], Ir-CPI [22], Desmolaris [13], and some artificial design Kunitz-type peptides Fasxiator_N17R,L19E_, BF9-N17K-L19D, DX-88mut, PN2KPI-M17D and WPK5-mut [14,20,23,24,27]. Nearly all of these inhibitors feature arginine or lysine at their P1 position, and BF9N17HD3 is the first potent and selective Kunitz-type XIa inhibitor with the unique P1 site histidine, which suggested that P1-site basic residues K, R or H and the P2′ site D or E might be a potential optimized combination for selective XIa inhibitors with the Kunitz-type scaffold, which provide a new clue for the design of specific XIa inhibitors [26,31,32]. However, no Kunitz-type XIa inhibitors without plasmin inhibitory activity have been reported. In our present work, we found that the P1 site histidine and the proper P2′ site combination might be a possible strategy to produce Kunitz-type XIa inhibitors without plasmin inhibitory activity (Figure 8C). To date, no Kunitz-type XIa peptide inhibitor has advanced into human clinical trials. All candidate molecules remain in the preclinical stage, including the krait-venom-derived Kunitz peptide Fasxiator, the *Deinagkistrodon acutus* venom Kunitz peptide DAKS1, the tick salivary gland Kunitz peptide Ir-CPI, and the human-derived Kunitz peptide PN2API. Two major factors account for this situation. The first is pharmacokinetic limitations. Although the Kunitz peptide scaffold exhibits relatively high stability, many natural or recombinant Kunitz peptides possess short plasma half-lives, in some cases merely several minutes. The only marketed drug with a Kunitz scaffold is ecallantide, a selective plasma kallikrein inhibitor indicated primarily for hereditary angioedema (HAE), which requires frequent subcutaneous administration. The second factor is pharmacological limitations. Naturally occurring canonical Kunitz molecules commonly exert cross-inhibitory activities against multiple serine proteases such as plasmin, XIIa, kallikrein and Xa. Obtaining a highly selective Kunitz peptide targeting solely XIa demands extensive site-directed mutagenesis of loop regions, substantially prolonging the research and development timeline. As a result, there have been no reports of highly selective Kunitz-type FXIa inhibitors devoid of activity against plasmin. The discovery of Kunitz-type XIa inhibitors with the unique P1 site histidine provides a new clue to enhance their selectivity of native Kunitz-type XIa inhibitors and their analogues and opens a new avenue for the development of novel anticoagulants targeting XIa.

## 4. Conclusions

In conclusion, we identified one XIa-targeting inhibitor with attenuated plasmin-inhibitory activity and five XIa inhibitors completely devoid of plasmin-inhibitory activity and highlight that the snake venom Kunitz-type scaffold serves as a promising molecular template for designing novel XIa-directed anticoagulants. In addition, the peptide engineering of the snake venom Kunitz-type scaffold also indicated that three basic amino acids, lysine, arginine and histidine, can exert distinct functional roles in different sites of Kunitz-type peptides.

## 5. Materials and Methods

### 5.1. Bioinformatics and Molecular Design of Snake Venom Kunitz-Peptide BF9 Analogues

The 3-D structures of BF9-N17H and its analogues were modelled with the SWISS-MODEL server by using the natural snake toxin scaffold BF9 as the template (PDB Code: 1JC6) [37,38]. According to the P2′ site that was reported to be associated with peptide selectivity [26], ten BF9-N17H analogues were designed via residue substitution at the P2′ position following canonical amino acid classification, such as polar amino acids S, N and Y, non-polar amino acids A and P, basic amino acids K, R and H, and acidic amino acids D and E. The complex structure of BF9N17HH10 and XIa was modelled using AlphaFold3.

### 5.2. Recombinant Plasmids and Peptides

Ten recombinant plasmids, pET-28a-BF9N17HA1, pET-28a-BF9N17HP2, pET-28a-BF9N17HD3, pET-28a-BF9N17HS4, pET-28a-BF9N17HN5, pET-28a-BF9N17HY6, pET-28a-BF9N17HE7, pET-28a-BF9N17HK8, pET-28a-BF9N17HR9, and pET-28a-BF9N17HH10, were constructed by the company and confirmed by DNA sequencing (GenScript Biotech Corporation, Nanjing, China). Ten recombinant BF9-N17H analogue peptides were produced following previously established protocols for the parental BF9-N17H peptide, with only minor adjustments to the pH of the refolding buffer that were used for refolding denatured peptides in the GSH-GSSG system that we built before [14,16,39,40]. The insoluble inclusion bodies were washed 2–3 times with the prepared washing buffer (1–2% Triton X-100 in the phosphate buffer) and denatured in a 5 mL prepared denaturation solution (6 M guanidinium-HCl, 0.1 M Tris-HCl with pH 8.0, 1 mM EDTA, and 30 mM reduced glutathione). Solubilized peptide material was diluted 100-fold into refolding buffer at varied pH values (0.2 M ammonium acetate, 0.2 mM oxidized glutathione and 0.5 M arginine) at 16 °C for about 24 h.

### 5.3. Serine Protease Inhibition Activity

Nine serine proteases and their corresponding fluorogenic substrates were used to test peptide activity by the Fluorescence Resonance Energy Transfer (FRET) strategy. The inhibitory experiments were done with the same method that we have described before, including the concentrations of enzymes and fluorogenic substrates [25,26,33]. To facilitate activity comparison, the final peptide concentration gradients were uniformly set as 0 nM, 78.125 nM, 156.25 nM, 312.5 nM, 625 nM, 1250 nM and 2500 nM. Peptides were serially diluted in assay buffer matched to each target enzyme. Fifty microliters of enzyme solution and 50 μL of serially diluted peptide were added to each well of a 96-well plate. The buffer was the negative control. Then, the substrate was added to another row, 96-well plates were placed on a shaking table at 37 °C, and they were incubated at 100 rpm for 30 min. At last, the 96-well plates were placed on the enzyme-linked immunosorbent assay (ELISA) reader and 100 μL substrate was added to each well; then, a wavelength of 405 nm was immediately detected.

The fluorogenic substrate of coagulation factor FXIa (Haematologic Technologies Inc., Shanghai, China) is S-2366 (Chromogenix, Shanghai, China). The fluorogenic substrate of coagulation factor FXIIa (Molecular Innovations, Shanghai, China) is S-2302 (Chromogenix, Shanghai, China). The fluorogenic substrate of coagulation factor FXa (Haematologic Technologies Inc., Shanghai, China) is S-2765 (Chromogenix, Shanghai, China). The fluorogenic substrate of coagulation factor FIIa (Chromogenix, Shanghai, China) is S-2238 (Chromogenix, Shanghai, China). The fluorogenic substrate of coagulation factor KLK (Enzyme Research Laboratories, Shanghai, China) is S-2302 (Chromogenix, Shanghai, China). The fluorogenic substrate of plasmin (Sigma, Shanghai, China) is S-2251 (Chromogenix, Shanghai, China). The fluorogenic substrate of trypsin (Sigma, Shanghai, China) is B3133 (Sigma, Shanghai, China). The fluorogenic substrate of chymotrypsin (Sigma, Shanghai, China) is S-7388 (Sigma, Shanghai, China). The fluorogenic substrate of elastase (Sigma, Shanghai, China) is S-4760 (Sigma, Shanghai, China).

### 5.4. Statistical Analysis

The results are expressed as the means ± SDs unless otherwise described. Curves of residual enzyme activity vs. inhibitor concentration were generated in SigmaPlot 12.5 software, and the IC_50_ was calculated according to the following equation (four parameter logistic curve): y = min + (max − min)/(1 + (x/IC_50_)^Hillslope^).

## Data Availability

The original contributions presented in this study are included in this article and the Supplementary Material. Further inquiries can be directed to the corresponding author.

## Supplementary Materials

The following supporting information can be downloaded at https://www.mdpi.com/article/10.3390/toxins18100449/s1, Figure S1: Purification of ten designed analogues by high-performance liquid chromatography (HPLC). Figure S2: SDS-PAGE analyses of ten BF9N17H analogues. Figure S3: Serine protease trypsin inhibitory activity of nine designed analogues. Figure S4: Serine protease chymotrypsin inhibitory activity of nine designed analogues. Figure S5: Serine protease elastase inhibitory activity of nine designed analogues. Figure S6: Serine protease IIa inhibitory activity of nine designed analogues. Figure S7: Serine protease Xa inhibitory activity of nine designed analogues. Figure S8: Serine protease KLK inhibitory activity of nine designed analogues. Figure S9: Serine protease XIIa inhibitory activity of nine designed analogues.

## References

[1] Moellmer S.A., Puy C., McCarty O.J.T. (2024). Biology of factor XI. Blood.

[2] Galli M., Occhipinti G., Ortega-Paz L., Franchi F., Rollini F., Brugaletta S., Capodanno D., Sciarretta S., Angiolillo D.J. (2024). Therapeutic Potential of FXI Inhibitors: Hype or Hope?. Drugs.

[3] Gailani D., Gruber A. (2024). Targeting factor XI and factor XIa to prevent thrombosis. Blood.

[4] Xie Z., Meng Z., Yang X., Duan Y., Wang Q., Liao C. (2023). Factor XIa Inhibitors in Anticoagulation Therapy: Recent Advances and Perspectives. J. Med. Chem..

[5] Harrington J., Piccini J.P., Alexander J.H., Granger C.B., Patel M.R. (2023). Clinical Evaluation of Factor XIa Inhibitor Drugs: JACC Review Topic of the Week. J. Am. Coll. Cardiol..

[6] Vlasuk G.P. (1993). Structural and functional characterization of tick anticoagulant peptide (TAP): A potent and selective inhibitor of blood coagulation factor Xa. Thromb. Haemost..

[7] Vlasuk G.P., Ramjit D., Fujita T., Dunwiddie C.T., Nutt E.M., Smith D.E., Shebuski R.J. (1991). Comparison of the in vivo anticoagulant properties of standard heparin and the highly selective factor Xa inhibitors antistasin and tick anticoagulant peptide (TAP) in a rabbit model of venous thrombosis. Thromb. Haemost..

[8] Waxman L., Smith D.E., Arcuri K.E., Vlasuk G.P. (1990). Tick anticoagulant peptide (TAP) is a novel inhibitor of blood coagulation factor Xa. Science.

[9] Rydel T.J., Ravichandran K.G., Tulinsky A., Bode W., Huber R., Roitsch C., Fenton J.W. (1990). The structure of a complex of recombinant hirudin and human alpha-thrombin. Science.

[10] Lombardi A., Nastri F., Della Morte R., Rossi A., De Rosa A., Staiano N., Pedone C., Pavone V. (1996). Rational design of true hirudin mimetics: Synthesis and characterization of multisite-directed alpha-thrombin inhibitors. J. Med. Chem..

[11] Johnson P.H. (1994). Hirudin: Clinical potential of a thrombin inhibitor. Annu. Rev. Med..

[12] Jmel M.A., Voet H., Araujo R.N., Tirloni L., Sa-Nunes A., Kotsyfakis M. (2023). Tick Salivary Kunitz-Type Inhibitors: Targeting Host Hemostasis and Immunity to Mediate Successful Blood Feeding. Int. J. Mol. Sci..

[13] Ma D., Mizurini D.M., Assumpcao T.C., Li Y., Qi Y., Kotsyfakis M., Ribeiro J.M., Monteiro R.Q., Francischetti I.M. (2013). Desmolaris, a novel factor XIa anticoagulant from the salivary gland of the vampire bat (*Desmodus rotundus*) inhibits inflammation and thrombosis in vivo. Blood.

[14] Ding L., Hao J., Luo X., Chen Z. (2018). Engineering varied serine protease inhibitors by converting P1 site of BF9, a weakly active Kunitz-type animal toxin. Int. J. Biol. Macromol..

[15] Ding L., Hao J., Luo X., Zhu W., Wu Z., Qian Y., Hu F., Liu T., Ruan X., Li S. (2018). The Kv1.3 channel-inhibitory toxin BF9 also displays anticoagulant activity via inhibition of factor XIa. Toxicon.

[16] Yang W., Feng J., Wang B., Cao Z., Li W., Wu Y., Chen Z. (2014). BF9, the first functionally characterized snake toxin peptide with Kunitz-type protease and potassium channel inhibiting properties. J. Biochem. Mol. Toxicol..

[17] Jiang S., Li Y., Zhang J., Jia W., Zheng Y., Jia Z., Yu C., Kong Y. (2024). Dual Inhibition of Factor XIIa and Factor XIa Produces a Synergistic Anticoagulant Effect. J. Cardiovasc. Pharmacol..

[18] Navaneetham D., Sinha D., Walsh P.N. (2010). Mechanisms and specificity of factor XIa and trypsin inhibition by protease nexin 2 and basic pancreatic trypsin inhibitor. J. Biochem..

[19] Wu W., Li H., Navaneetham D., Reichenbach Z.W., Tuma R.F., Walsh P.N. (2012). The kunitz protease inhibitor domain of protease nexin-2 inhibits factor XIa and murine carotid artery and middle cerebral artery thrombosis. Blood.

[20] Zheng Y.Z., Ji X.R., Liu Y.Y., Jiang S., Yu X.Y., Jia Z.P., Zhao Y., Zhang J.Q., Zhang J.L., Kong Y. (2021). WPK5, a Novel Kunitz-Type Peptide from the Leech *Whitmania pigra* Inhibiting Factor XIa, and Its Loop-Replaced Mutant to Improve Potency. Biomedicines.

[21] Jia Z., Liu Y., Ji X., Zheng Y., Li Z., Jiang S., Li H., Kong Y. (2021). DAKS1, a Kunitz Scaffold Peptide from the Venom Gland of *Deinagkistrodon acutus* Prevents Carotid-Artery and Middle-Cerebral-Artery Thrombosis via Targeting Factor XIa. Pharmaceuticals.

[22] Decrem Y., Rath G., Blasioli V., Cauchie P., Robert S., Beaufays J., Frere J.M., Feron O., Dogne J.M., Dessy C. (2009). Ir-CPI, a coagulation contact phase inhibitor from the tick *Ixodes ricinus*, inhibits thrombus formation without impairing hemostasis. J. Exp. Med..

[23] Sun F., Wang W., Li Z., Li Y., Guo W., Kong Y. (2024). Design, expression and biological evaluation of DX-88mut as a novel selective factor XIa inhibitor for antithrombosis. Bioorganic Chem..

[24] Eltringham-Smith L.J., Bhakta V., Sheffield W.P. (2021). Selection and in vitro and in vivo characterization of a Kunitz protease inhibitor domain of protease nexin 2 variant that inhibits factor XIa without inhibiting plasmin. J. Biotechnol..

[25] Mishra M. (2020). Evolutionary Aspects of the Structural Convergence and Functional Diversification of Kunitz-Domain Inhibitors. J. Mol. Evol..

[26] Navaneetham D., Wu W., Li H., Sinha D., Tuma R.F., Walsh P.N. (2013). P1 and P2′ site mutations convert protease nexin-2 from a factor XIa inhibitor to a plasmin inhibitor. J. Biochem..

[27] Chen W., Carvalho L.P., Chan M.Y., Kini R.M., Kang T.S. (2015). Fasxiator, a novel factor XIa inhibitor from snake venom, and its site-specific mutagenesis to improve potency and selectivity. J. Thromb. Haemost..

[28] Pham P.N., Huliciak M., Biedermannova L., Cerny J., Charnavets T., Fuertes G., Herynek S., Kolarova L., Kolenko P., Pavlicek J. (2021). Protein Binder (ProBi) as a New Class of Structurally Robust Non-Antibody Protein Scaffold for Directed Evolution. Viruses.

[29] Yasui N., Nakamura K., Yamashita A. (2021). A sweet protein monellin as a non-antibody scaffold for synthetic binding proteins. J. Biochem..

[30] Vazquez-Lombardi R., Phan T.G., Zimmermann C., Lowe D., Jermutus L., Christ D. (2015). Challenges and opportunities for non-antibody scaffold drugs. Drug Discov. Today.

[31] Vadivel K., Kumar Y., Ogueli G.I., Ponnuraj S.M., Wongkongkathep P., Loo J.A., Bajaj M.S., Bajaj S.P. (2016). S2′-subsite variations between human and mouse enzymes (plasmin, factor XIa, kallikrein) elucidate inhibition differences by tissue factor pathway inhibitor -2 domain1-wild-type, Leu17Arg-mutant and aprotinin. J. Thromb. Haemost..

[32] Bajaj M.S., Ogueli G.I., Kumar Y., Vadivel K., Lawson G., Shanker S., Schmidt A.E., Bajaj S.P. (2011). Engineering kunitz domain 1 (KD1) of human tissue factor pathway inhibitor-2 to selectively inhibit fibrinolysis: Properties of KD1-L17R variant. J. Biol. Chem..

[33] Hsueh H.T., Chou R.T., Rai U., Liyanage W., Kim Y.C., Appell M.B., Pejavar J., Leo K.T., Davison C., Kolodziejski P. (2023). Machine learning-driven multifunctional peptide engineering for sustained ocular drug delivery. Nat. Commun..

[34] Zhang H., Lin J.J., Xie Y.K., Song X.Z., Sun J.Y., Zhang B.L., Qi Y.K., Xu Z.Z., Yang F. (2023). Structure-guided peptide engineering of a positive allosteric modulator targeting the outer pore of TRPV1 for long-lasting analgesia. Nat. Commun..

[35] Imai M., Colas K., Suga H. (2024). Protein Grafting Techniques: From Peptide Epitopes to Lasso-Grafted Neobiologics. ChemPlusChem.

[36] Navaneetham D., Jin L., Pandey P., Strickler J.E., Babine R.E., Abdel-Meguid S.S., Walsh P.N. (2005). Structural and mutational analyses of the molecular interactions between the catalytic domain of factor XIa and the Kunitz protease inhibitor domain of protease nexin 2. J. Biol. Chem..

[37] Chen C., Hsu C.H., Su N.Y., Lin Y.C., Chiou S.H., Wu S.H. (2001). Solution structure of a Kunitz-type chymotrypsin inhibitor isolated from the elapid snake *Bungarus fasciatus*. J. Biol. Chem..

[38] Waterhouse A., Bertoni M., Bienert S., Studer G., Tauriello G., Gumienny R., Heer F.T., de Beer T.A.P., Rempfer C., Bordoli L. (2018). SWISS-MODEL: Homology modelling of protein structures and complexes. Nucleic Acids Res..

[39] Chen Z., Luo F., Feng J., Yang W., Zeng D., Zhao R., Cao Z., Liu M., Li W., Jiang L. (2013). Genomic and structural characterization of Kunitz-type peptide LmKTT-1a highlights diversity and evolution of scorpion potassium channel toxins. PLoS ONE.

[40] Chen Z.Y., Hu Y.T., Yang W.S., He Y.W., Feng J., Wang B., Zhao R.M., Ding J.P., Cao Z.J., Li W.X. (2012). Hg1, novel peptide inhibitor specific for Kv1.3 channels from first scorpion Kunitz-type potassium channel toxin family. J. Biol. Chem..

