# Supplementary material for: P2′ Site Scanning of Snake Kunitz-Type Toxin Analogue BF9-N17H with Unique P1 Site Histidine Highlights Novel XIa Inhibitory Peptides Without Plasmin Inhibitory Activity
Source: Toxins (Basel). 2026 Oct 15;18(10):449. doi: 10.3390/toxins18100449 (PMC13709558; doi:10.3390/toxins18100449)
Supplement: Supplementary file 1 [file toxins-18-00449-s001.zip › toxins-4491410-supplementary.pdf]

Supplementary Materials: P2′ Site Scanning of Snake Kunitz-type Toxin Analogue BF9-N17H with Unique P1 Site Histidine Highlights Novel XIa Inhibitory Peptides without Plasmin Inhibitory Activity

Luyao Wang [†], Li Ding [†], Tianxiang Qin [†], Xinyu Liu, Chenglan Lou, Hongyi Luo, Fang Sun, Chenhu Qin, Xiangdong Ye, Xudong Luo* and Zongyun Chen *

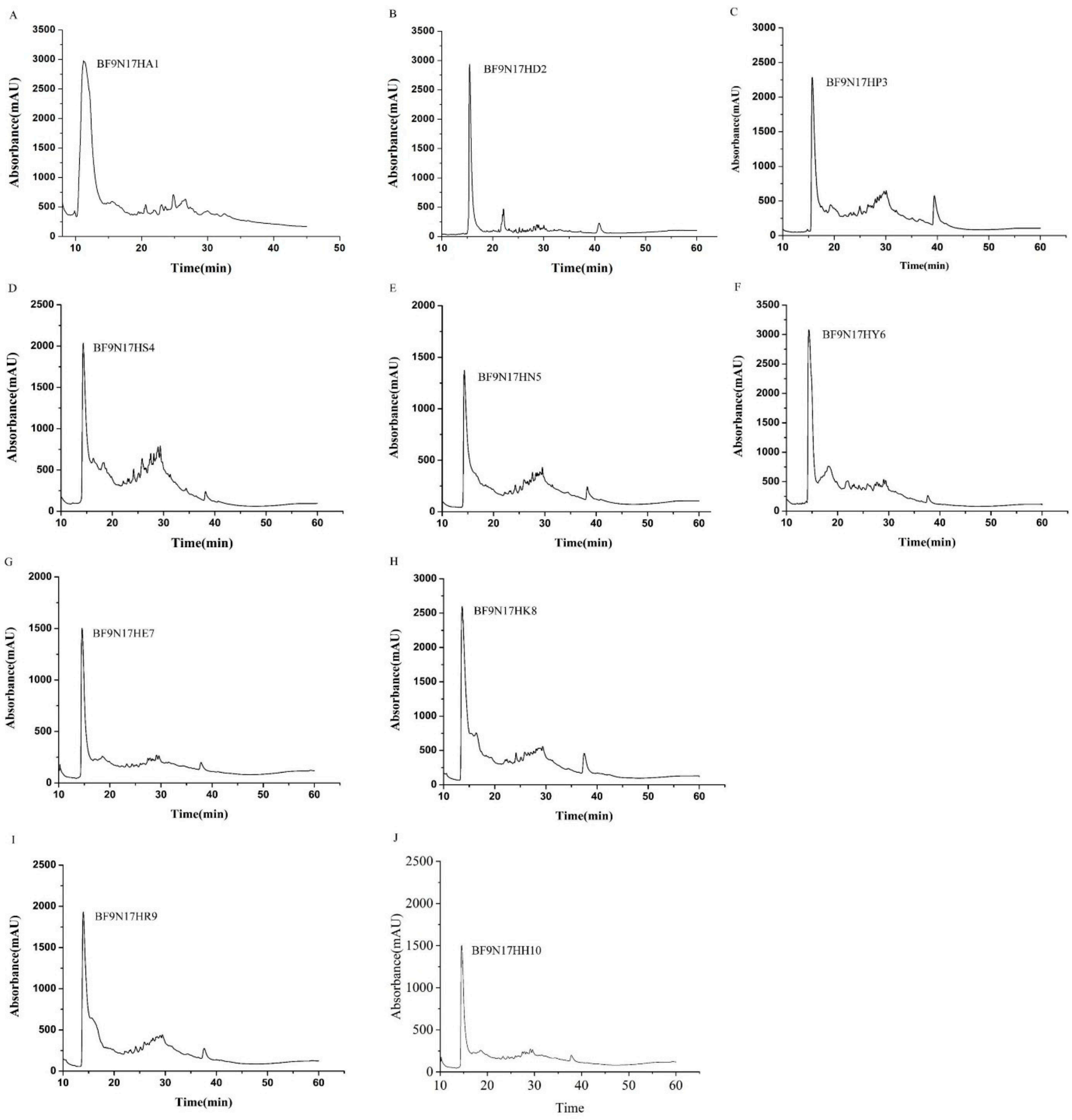

Figure S1. Purification of ten designed analogues by high performance liquid chromatography (HPLC). A, Purification of BF9N17HA1; B, Purification of BF9N17HD2; C, Purification of BF9N17HP3; D, Purification of BF9N17HS4; E, Purification of BF9N17HN5; F, Purification of BF9N17HY6; G, Purification of BF9N17HE7; H, Purification of BF9N17HK8; I, Purification of BF9N17HR9; J, Purification of BF9N17HH10.

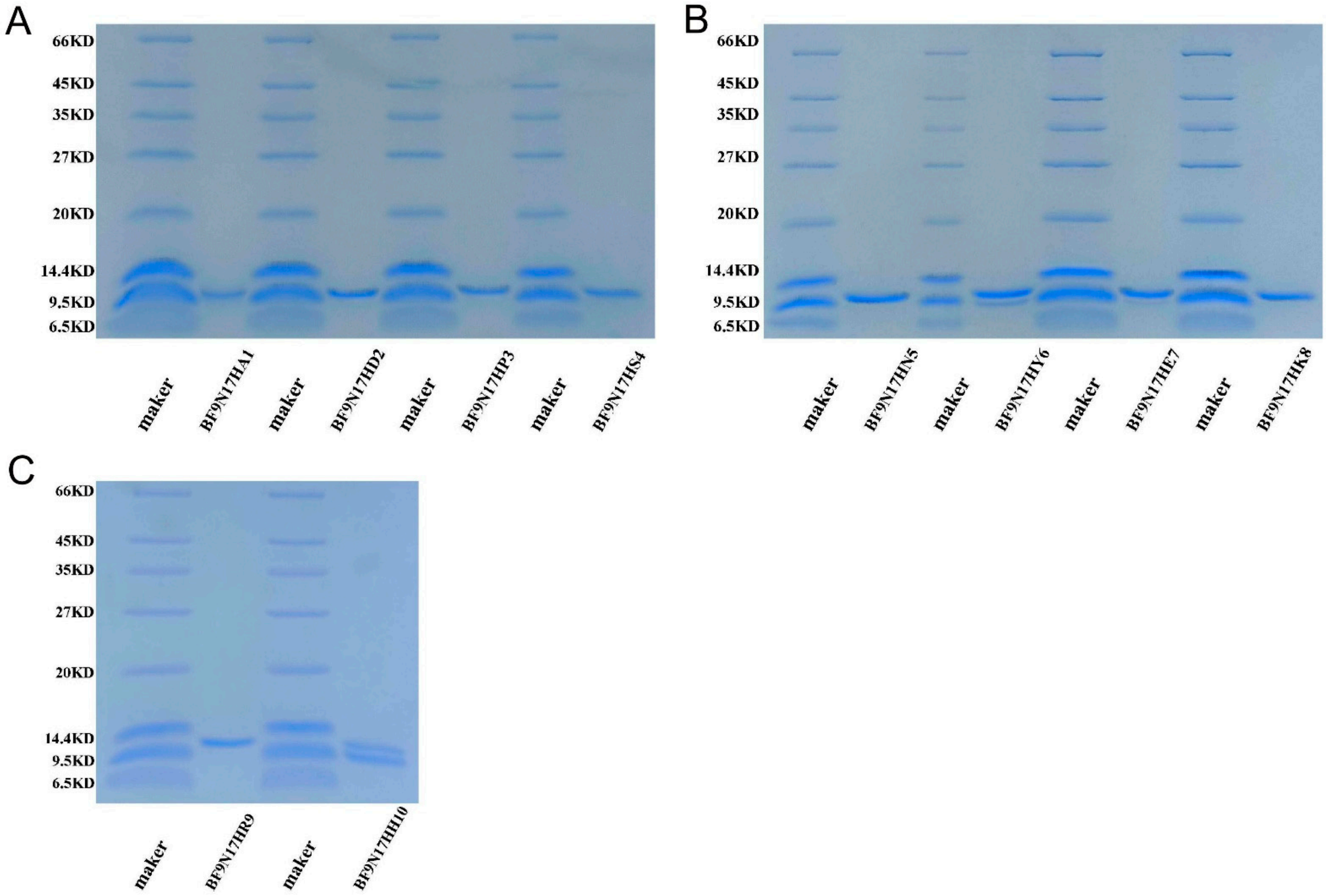

Figure S2. SDS-PAGE analyses of ten BF9N17H analogues. A, SDS-PAGE analyses of BF9N17HA1, BF9N17HD2, BF9N17HP3 and BF9N17HS4; B, SDS-PAGE analyses of BF9N17HN5, BF9N17HY6, BF9N17HE7 and BF9N17HK8; C, SDS-PAGE analyses of BF9N17HR9 and BF9N17HH10.

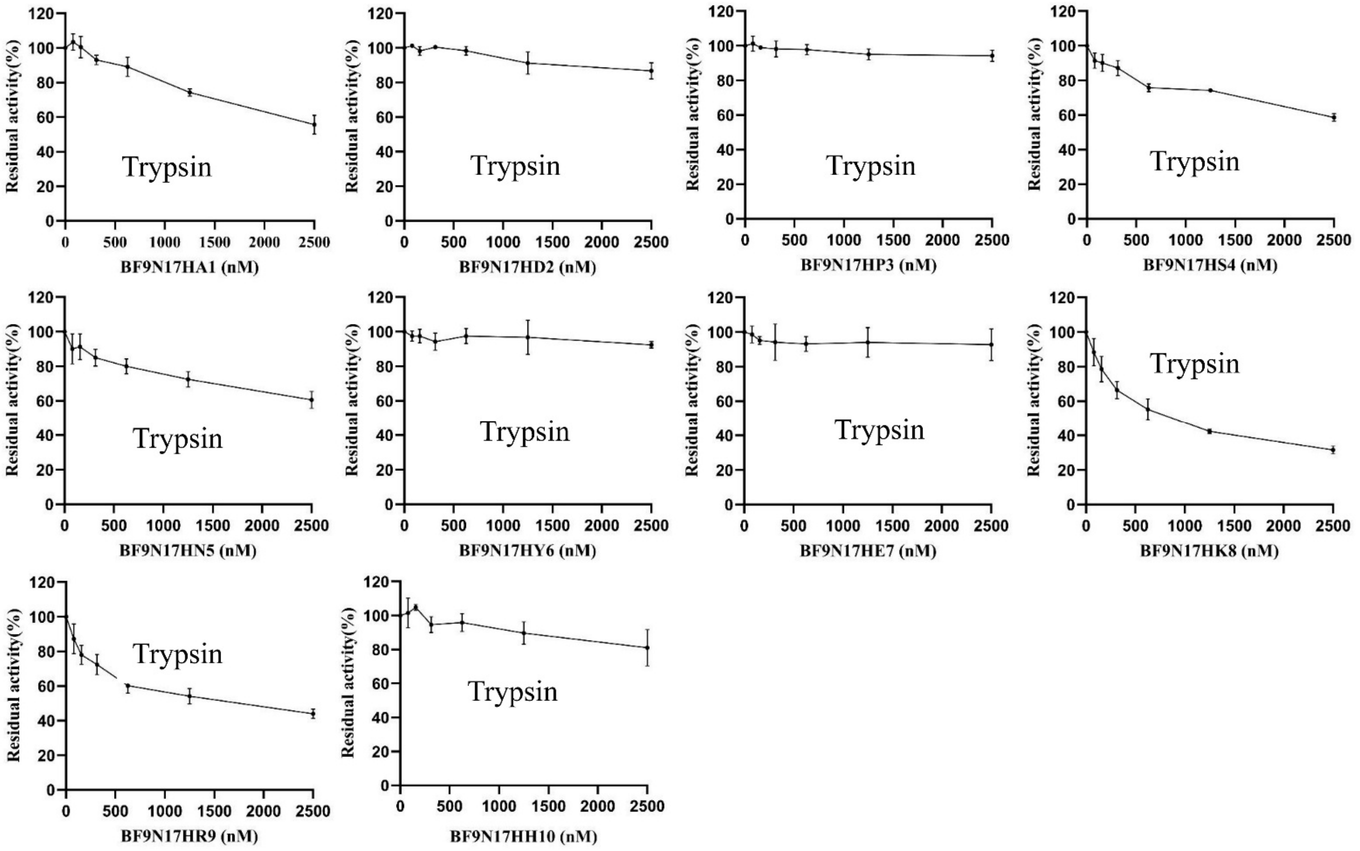

Figure S3. Serine protease trypsin inhibitory activity of nine designed analogues. A, Trypsin inhibitory activity of BF9N17HA1; B, Trypsin inhibitory activity of BF9N17HD2; C, Trypsin inhibitory activity of BF9N17HP3; D, Trypsin inhibitory activity of BF9N17HS4; E, Trypsin inhibitory activity of BF9N17HH10; F, Trypsin inhibitory activity of BF9N17HY6; G, Trypsin inhibitory activity of BF9N17HE7; H, Trypsin inhibitory activity of BF9N17HK8; I, Trypsin inhibitory activity of BF9N17HR9, J, Trypsin inhibitory activity of BF9N17HH10.

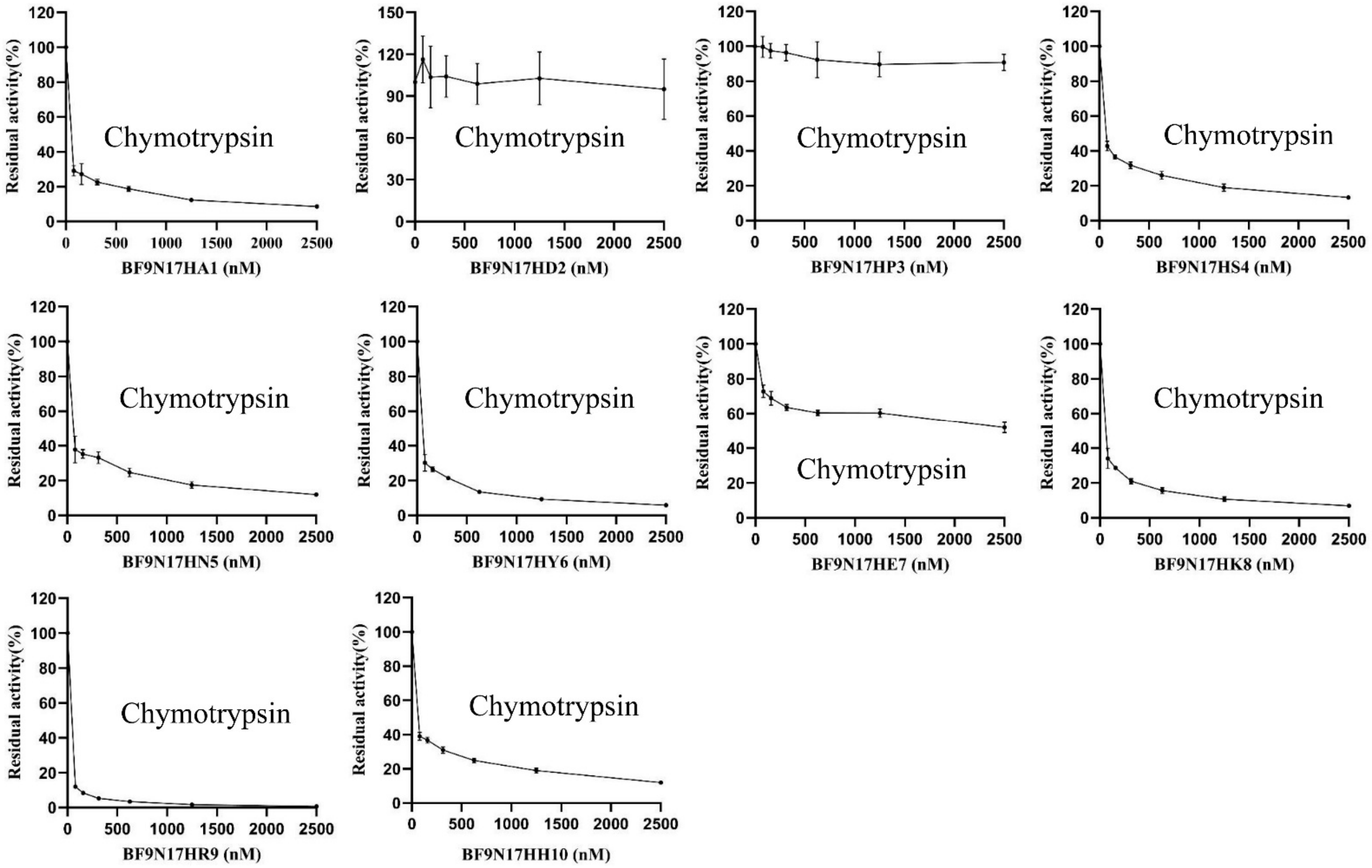

Figure S4. Serine protease chymotrypsin inhibitory activity of nine designed analogues. A, Chymotrypsin inhibitory activity of BF9N17HA1; B, Chymotrypsin inhibitory activity of BF9N17HD2; C, Chymotrypsin inhibitory activity of BF9N17HP3; D, Chymotrypsin inhibitory activity of BF9N17HS4; E, Chymotrypsin inhibitory activity of BF9N17HN5; F, Chymotrypsin inhibitory activity of BF9N17HY6; G, Chymotrypsin inhibitory activity of BF9N17HE7; H, Chymotrypsin inhibitory activity of BF9N17HK8; I, Chymotrypsin inhibitory activity of BF9N17HR9, J, Chymotrypsin inhibitory activity of BF9N17HH10.

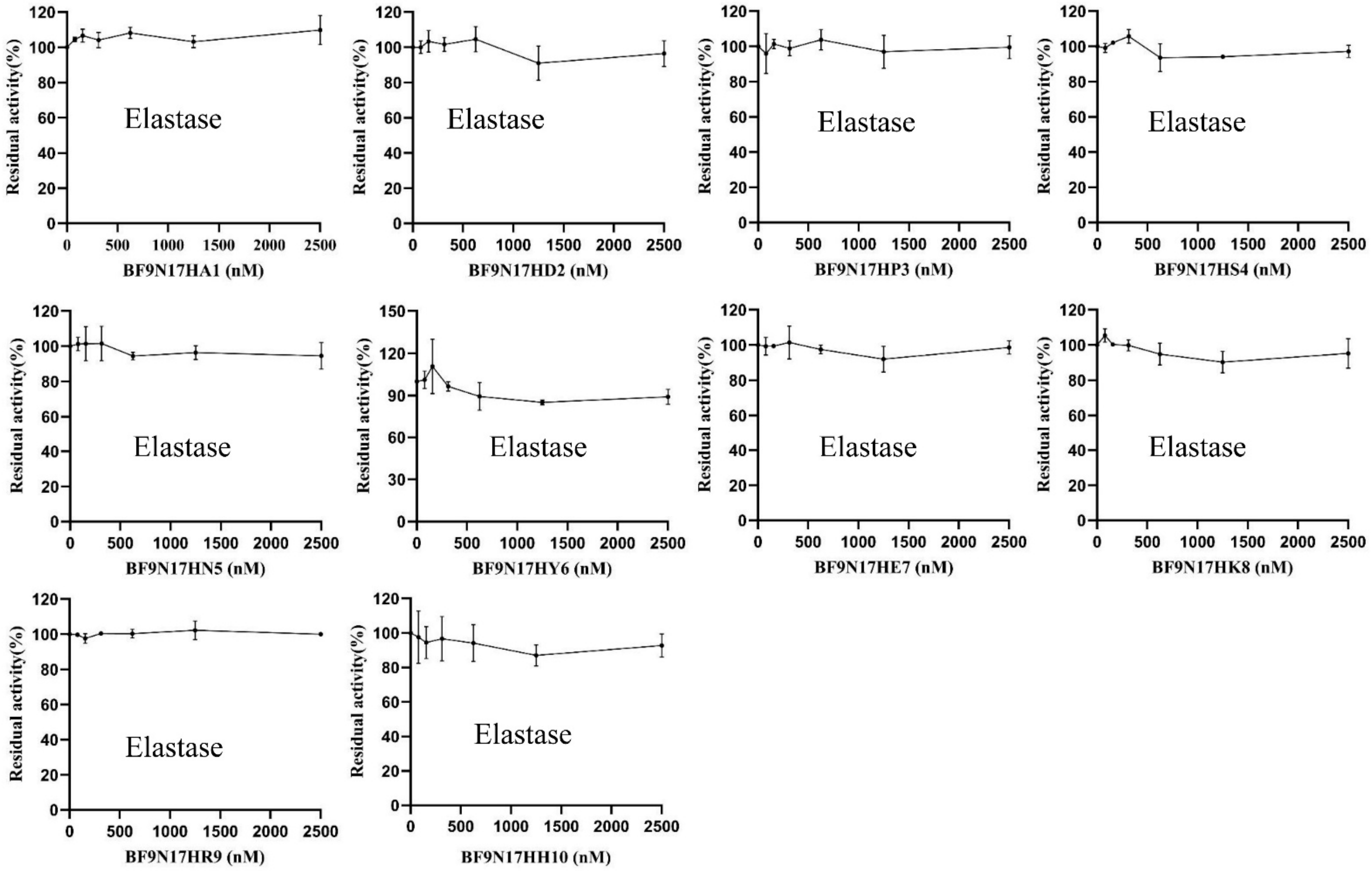

Figure S5. Serine protease elastase inhibitory activity of nine designed analogues. A, Elastase inhibitory activity of BF9N17HA1; B, Elastase inhibitory activity of BF9N17HD2; C, Elastase inhibitory activity of BF9N17HP3; D, Elastase inhibitory activity of BF9N17HS4; E, Elastase inhibitory activity of BF9N17HN5; F, Elastase inhibitory activity of BF9N17HY6; G, Elastase inhibitory activity of BF9N17HE7; H, Elastase inhibitory activity of BF9N17HK8; I, Elastase inhibitory activity of BF9N17HR9; J, Elastase inhibitory activity of BF9N17HH10.

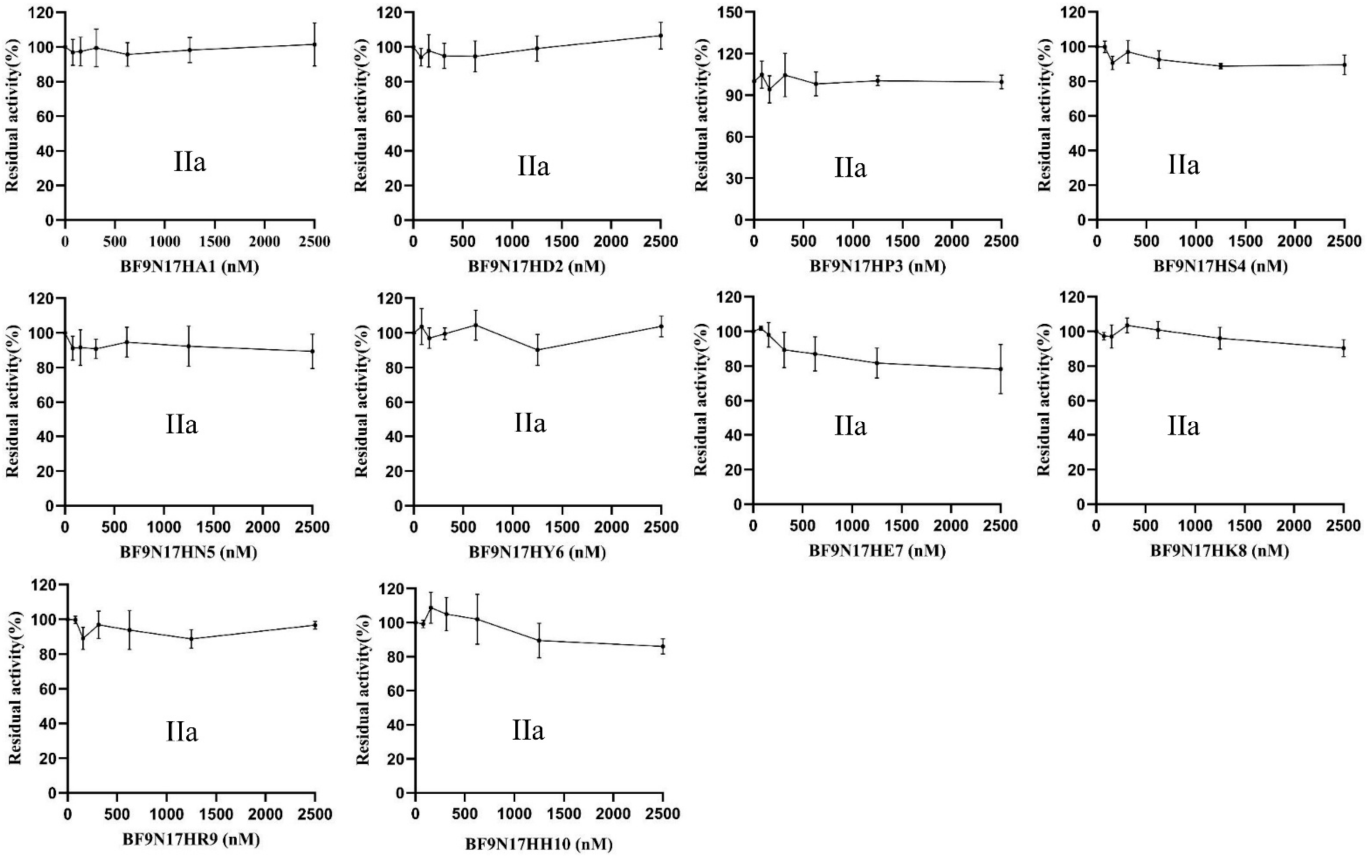

Figure S6. Serine protease IIa inhibitory activity of nine designed analogues. A, IIa inhibitory activity of BF9N17HA1; B, IIa inhibitory activity of BF9N17HD2; C, IIa inhibitory activity of BF9N17HP3; D, IIa inhibitory activity of BF9N17HS4; E, IIa inhibitory activity of BF9N17HN5; F, IIa inhibitory activity of BF9N17HY6; G, IIa inhibitory activity of BF9N17HE7; H, IIa inhibitory activity of BF9N17HK8; I, IIa inhibitory activity of BF9N17HR9; J, IIa inhibitory activity of BF9N17HH10.

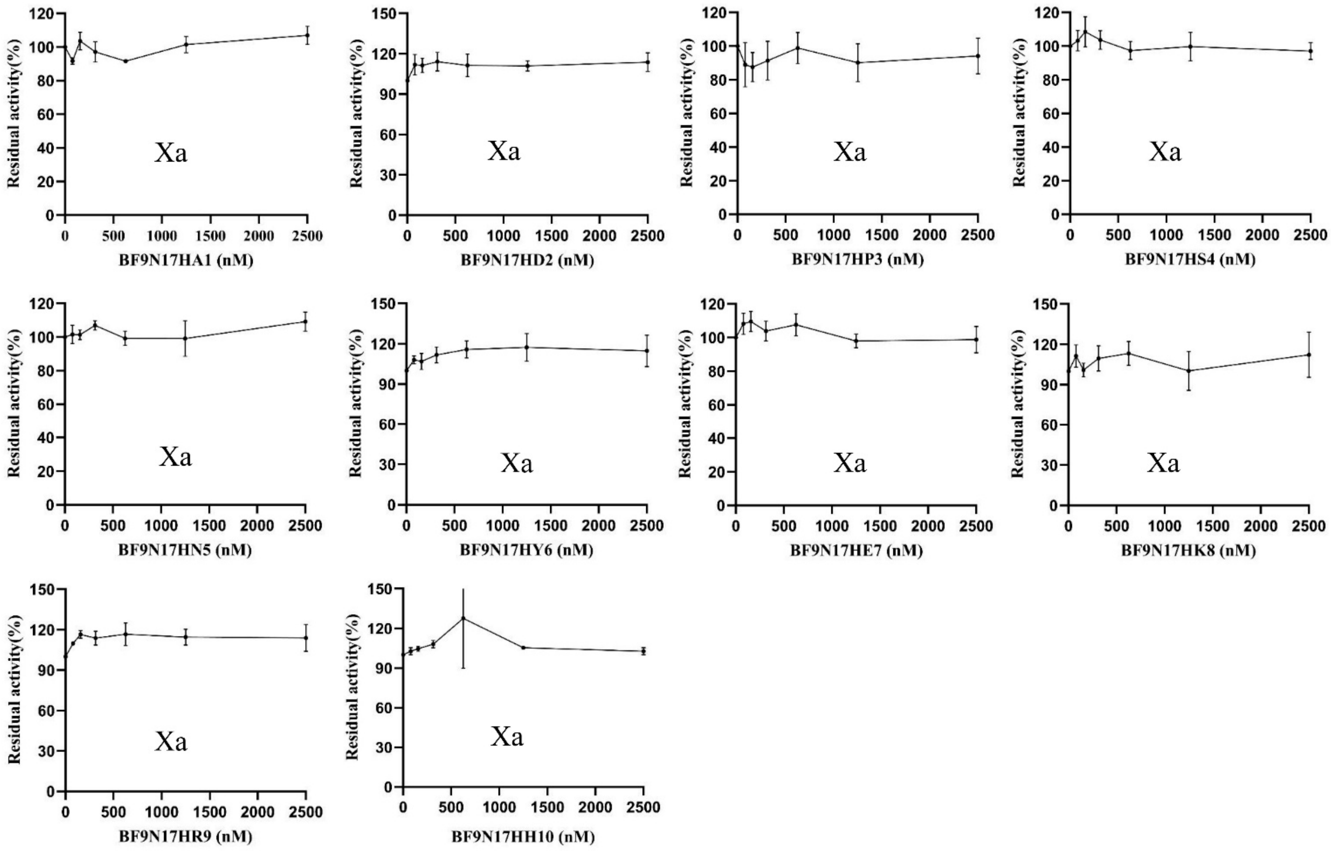

Figure S7. Serine protease Xa inhibitory activity of nine designed analogues. A, Xa inhibitory activity of BF9N17HA1; B, Xa inhibitory activity of BF9N17HD2; C, Xa inhibitory activity of BF9N17HP3; D, Xa inhibitory activity of BF9N17HS4; E, Xa inhibitory activity of BF9N17HN5; F, Xa inhibitory activity of BF9N17HY6; G, Xa inhibitory activity of BF9N17HE7; H, Xa inhibitory activity of BF9N17HK8; I, Xa inhibitory activity of BF9N17HR9, J, Xa inhibitory activity of BF9N17HH10.

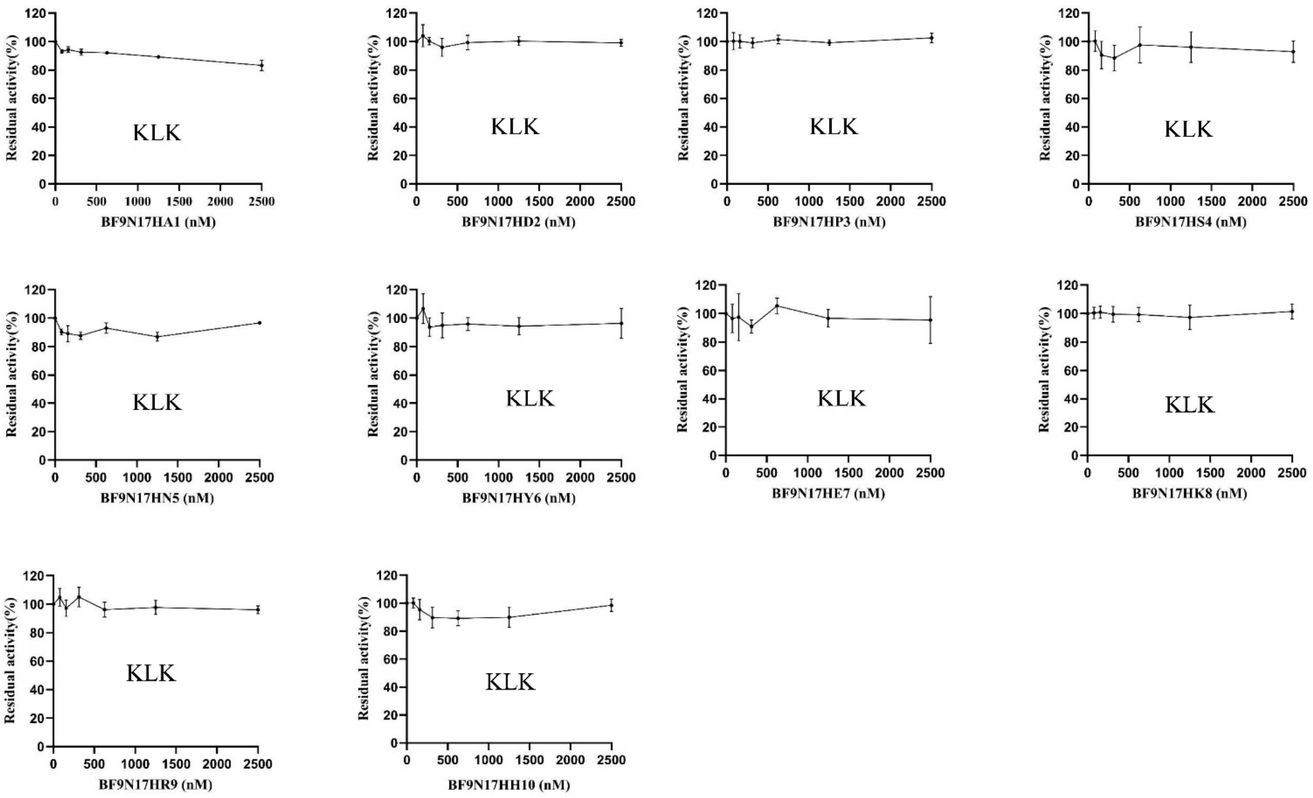

Figure S8. Serine protease KLK inhibitory activity of nine designed analogues. A, KLK inhibitory activity of BF9N17HA1; B, KLK inhibitory activity of BF9N17HD2; C, KLK inhibitory activity of BF9N17HP3; D, KLK inhibitory activity of BF9N17HS4; E, KLK inhibitory activity of BF9N17HN5; F, KLK inhibitory activity of BF9N17HY6; G, KLK inhibitory activity of BF9N17HE7; H, KLK inhibitory activity of BF9N17HK8; I, KLK inhibitory activity of BF9N17HR9; J, KLK inhibitory activity of BF9N17HH10.

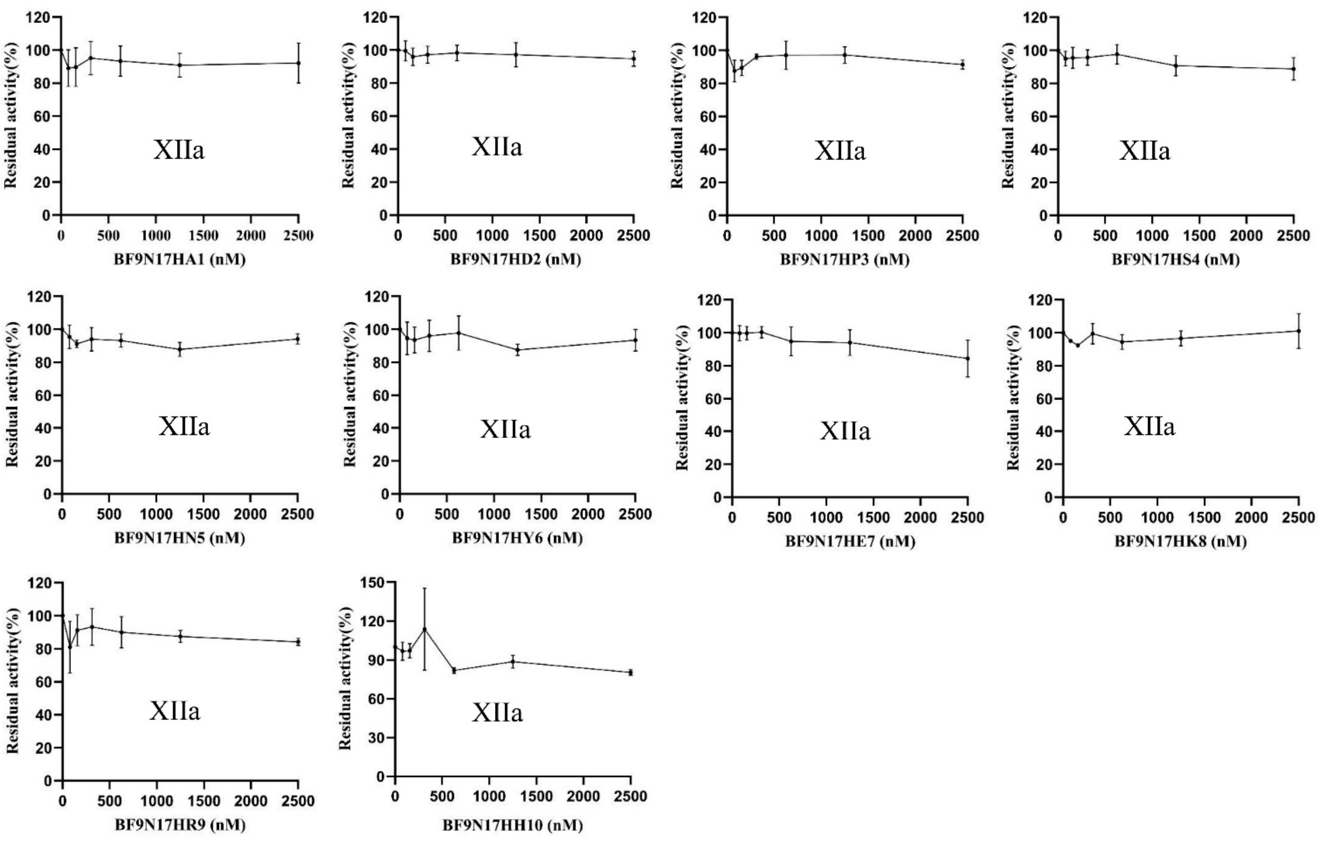

Figure S9. Serine protease XIIa inhibitory activity of nine designed analogues. A, XIIa inhibitory activity of BF9N17HA1; B, XIIa inhibitory activity of BF9N17HD2; C, XIIa inhibitory activity of BF9N17HP3; D, XIIa inhibitory activity of BF9N17HS4; E, XIIa inhibitory activity of BF9N17HN5; F, XIIa inhibitory activity of BF9N17HY6; G, XIIa inhibitory activity of BF9N17HE7; H, XIIa inhibitory activity of BF9N17HK8; I, XIIa inhibitory activity of BF9N17HR9; J, XIIa inhibitory activity of BF9N17HH10.
